# Research activity and capability in the European reference network MetabERN

**DOI:** 10.1186/s13023-019-1091-8

**Published:** 2019-05-29

**Authors:** Jean-Michel Heard, Cinzia Bellettato, Corine van Lingen, Maurizio Scarpa, the MetabERN collaboration group, François-Guillaume Debray, Marie-Cécile Nassogne, Rudy van Coster, Linda De Meirleir, François Eyskens, Eva Morava, Ivo Baric, Viktor Kozich, Allan Meldgaard Lund, Dominique Germain, Nadia Belmatoug, Nathalie Guffon, Philippe Labrune, Laurent Gouya, Pascale de Lonlay, Manuel Schiff, Dries Dobbelaere, Brigitte Chabrol, Anihb Martin Das, Maurizio Scarpa, Ute Spiekerkoetter, Frank Rutsch, Ursula Ploeckinger, Klaus Mohnike, Andreas Hahn, Stefan Kölker, Kurt Ullrich, István Balogh, Bruno Bembi, Maria Alice Donati, Serena Gasperini, Giancarlo Parenti, Alessandro Salviati, Carlo-Dionisi Vici, Maja di Rocco, Graziella Cefalo, Alberto Burlina, Giovanni Ceccarini, Antonio Federico, Ans van der Ploeg, Maria-Estela Rubio-Gozalbo, Francian van Spronsen, Gepke Visser, Annet Bosch, Trine Tangeraas, Sverre Sanderberg, Beata Kieć-Wilk, Ana-Maria Simões Mendes Gaspar, Esmeralda Martins, Esmeralda-Maria Ferreira Rodrigues Silva, Luísa-Maria de Abreu Freire Diogo Matos, Olga Azevedo, Mojca-Zerjav Tansek, Maria-Luz Couce-Pico, Angeles Garcia Cazorla, Luis Aldámiz-Echevarría Azuara, Mireia del Toro-Riera, Svetlana Lajic, Niklas Darin, Patrick Deegan, Suresh Vijaym, Efstathia Chronopoulou, Simon Jones, Anupanm Chakrapani, Tarekegn Hiwot

**Affiliations:** 1 MetabERN, Regional Coordinating Center for Rare Diseases, Udine University Hospital, Piazzale Santa Maria della Misericordia, 15, 33100 Udine, Italy; 2 Cinzia Bellettato, MetabERN, Udine, Italy; 3 Corine van Lingen, MetabERN, Udine, Italy; 4 Maurizio Scarpa, MetabERN, Udine, Italy

**Keywords:** European reference Centres, Hereditary metabolic diseases, Medical research activity, Multidisciplinary research

## Abstract

**Background:**

MetabERN is one of the 24 European Reference Networks created according to the European Union directive 2011/24/EU on patient’s rights in cross border healthcare. MetabERN associates 69 centres in 18 countries, which provide care for patients with Hereditary Metabolic Diseases, and have the mission to reinforce research and provide training for health professionals in this field. MetabERN performed a survey in December 2017 with the aim to produce an overview documenting research activities and potentials within the network. As the centres are multidisciplinary, separated questionnaires were sent to the clinical, university and laboratory teams. Answers were received from 52 out of the 69 centres of the network, covering 16 countries. A descriptive analysis of the information collected is presented.

**Results:**

The answers indicate a marked interest of the respondents for research, who expressed high motivation and commitment, and estimated that the conditions to do research in their institution were mostly satisfactory. They are active in research, which according to several indicators, is competitive and satisfies standards of excellence, as well as the education programs offered in the respondent’s universities. Research in the centres is primarily performed in genetics, pathophysiology, and epidemiology, and focuses on issues related to diagnosis. Few respondents declared having activity in human and social sciences, including research on patient’s quality of life, patient’s awareness, or methods for social support. Infrastructures offering services for medical research were rarely known and used by respondents, including national and international biobanking platforms. In contrast, respondents often participate to patient registries, even beyond their specific field of interest.

**Conclusions:**

Taken as a whole, these results provide an encouraging picture of the research capacities and activities in the MetabERN network, which, with respect to the number and representativeness of the investigated centres, gives a comprehensive picture of research on Hereditary Metabolic Diseases in Europe, as well as the priorities for future actions. Marginal activity in human and social sciences points out the limited multidisciplinary constitution of the responding teams with possible consequences on their current capability to participate to patient’s empowerment programs and efficiently collaborate with patient’s advocacy groups.

**Electronic supplementary material:**

The online version of this article (10.1186/s13023-019-1091-8) contains supplementary material, which is available to authorized users.

## Background

The European Union (EU) directive 2011/24/EU on patient’s rights in cross-border healthcare enables patients to be reimbursed for treatment in another EU Member State [1]. It facilitates the access of patients to information on healthcare and thus increases their treatment options. It provides patients with the best treatment and advice available in the EU for their specific condition, and provides healthcare professionals (HCPs) with access to a highly specialized pool of colleagues from all over Europe. The directive is especially relevant for rare and complex diseases, which affect around 30 million people in the EU. Article 12 of the directive created the European Reference Networks (ERNs) between HCPs and centres of expertise in the Member States in the area of rare diseases [2, 3]. ERNs are intended to concentrate resources, to pool knowledge and spread best practices, to exploit innovations in medical science and health technologies, and to improve diagnosis and the delivery of healthcare especially in Member States with an insufficient number of patients with a particular medical condition or lacking technology or expertise to provide highly specialized services. In addition, ERNs have the mission to reinforce research in all its aspects, clinical, preclinical, epidemiological, diagnostic, therapeutics, social sciences [4], and to provide training for health professionals. Twenty-four ERNs working on a range of thematic issues became operational in 2017. They comprise more than 900 highly specialized healthcare units located in 313 hospitals in 25 Member States (plus Norway).

MetabERN (https://metab.ern-net.eu/) is the network specifically dedicated to Hereditary Metabolic Diseases (HMDs). There are more than 1000 rare HMDs [5] belonging to 7 subgroups: 1) Amino and organic acids related disorders (AOA); 2) Disorders of pyruvate metabolism, Krebs cycle defects, mitochondrial oxidative phosphorylation disorders, disorders of thiamine transport and metabolism (PM-MD); 3) Carbohydrate, fatty acid oxidation and ketone bodies disorders (C-FAO); 4) Lysosomal disorders (LSD); 5) Peroxisomal and lipid related disorders (PD); 6) Congenital disorders of glycosylation and disorders of intracellular trafficking (CDG); and 7) Disorders of neuromodulators and small molecules (NOMS). MetabERN associates 69 centres in 17 Member States plus Norway (see the list in Additional file 1), mostly belonging to University hospitals, in which 1671 professionals follow more than 40,000 patients, of whom two-third are children.

As a first step in implementing its research mission, MetabERN performed a survey in December 2017. The primary aim was an overview documenting research activities and potentials within the 69 centres of the network. Since the MetabERN centres comprise multidisciplinary teams, declarations were collected by proper figures such as University professors, laboratory heads and heads of research units, according to the need. The secondary aim of the study was to identify the strengths and the weaknesses of research on HMDs in the network. With respect to the size and presumed representativeness of the investigated centres, the survey was intended to contribute to mapping research activity in the field of HMDs in the EU.

## Results

We received answers from 52 out of the 69 centres of the network, covering 16 out of the 18 countries participating to MetabERN (Additional file 1: Table S1). Answers were not uniformly received from the three contacted groups of each centre (the clinical group, the university group, and the laboratory group, see the methods section) and some groups sent several answers (Additional file 1: Table S1). We received a total of 148 responses to the 207 invitations sent to participate to the survey (66%). They included 52, 52 and 44 responses for the clinical, university and laboratory groups, respectively. The questions and multiple answers choice questionnaires are shown as Additional file 1. However, not all respondents completed the entire questionnaire. The analysis of each individual question was performed according to the number of complete responses to each question, which varies from one question to another.

### Research and teaching in the host institution

Questions related to personal research and teaching activity, and research and teaching in the host institution, were answered by 113 participants (clin. 35, univ. 43, lab. 35). Almost all respondents were active in research (clin. 97%, univ. 85%, lab. 100%) and had published at least one article in an ISI-referenced journal during the last 3 years. These publications associated international collaborators (> 40%) and/or collaborators from other national institutions (> 50%).

Investigators in the clinical group answered questions about financial support (*n* = 43) indicating that they receive national (40/43) and/or international (30/43) grants, to which many of them have applied as the study coordinator (international: 60%, national: 90%, *n* = 43). Institutional funding by the government is another important resource (38/43), as well as contracts with the industry (37/43). Collaborative research with the industry is a common practice for investigators in the clinical group (77% are engaged with an international company, 65% with a national company, *n* = 35), whereas this is more occasional for investigators in the university group (42% international and national, *n* = 43).

Almost all participants declared that their implication in research satisfies personal motivation to get more expertise. The main perceived barrier to do research is unanimously the lack of time due to high clinical workload, followed by insufficient funding. However, centres in which rotational positions for clinicians or researchers are organized remain minority (40 and 37% for clinicians and researchers, respectively). Nevertheless, physicians are involved in research in 94% of the clinical groups (most often more than 4 clinicians in the group participate, *n* = 37), as compared to 72% for the professors, 68% for the PhDs and 50% for the nurses (on average, one to three persons for each of these categories are involved).

Participants in the clinical group estimate that research performed in their institution reaches international (36/37) and national (36/37) standards with respect to excellence, that it contributes to local/regional development (33/37), to the quality of teaching (33/37), and increases the attractiveness of the institution (35/37), the contacts with industry (32/37), as well as the networking activities (33/37) and the implementation of quality assurance processes (33/37).

Answers received from the clinical departments (*n* = 35) also indicate that their host institutions award doctorates (29/35), some offering teaching in English (19/35) and theses defended in English (21/35), with interdisciplinary PhD programs (29/35) comprising mobility (28/35), student exchanges (21/35) and sometime international joint doctoral programs (16/35).

### Research activity of the centres

**Fig. 1:**
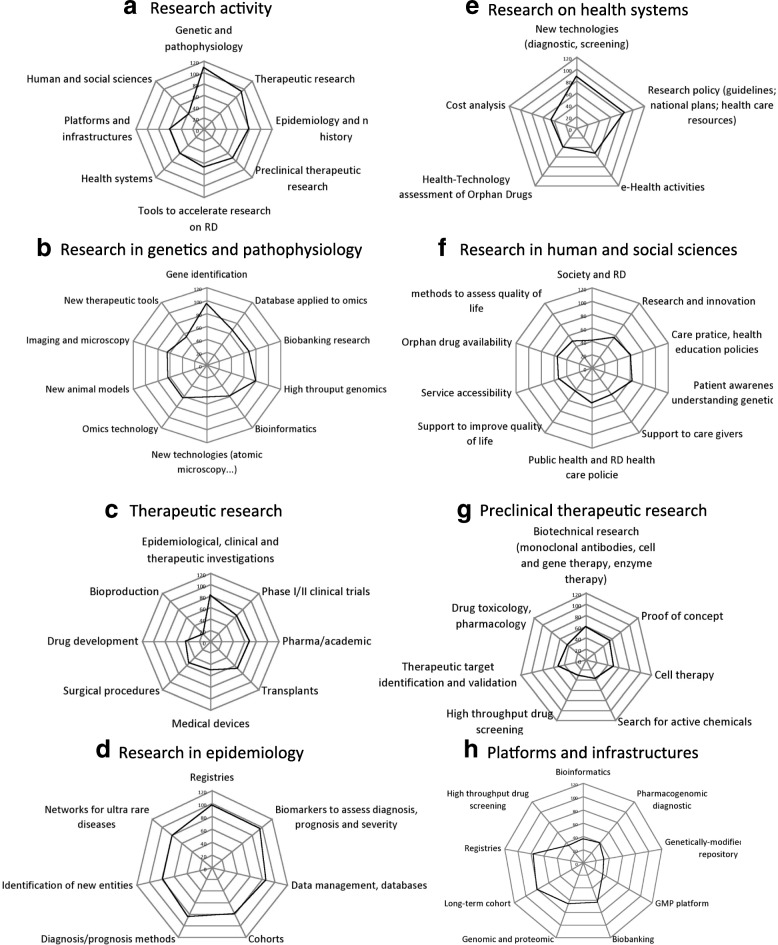
Numbers of responses indicating « high », or « rather high » activity in the indicated field out of 122 answers

The survey collected 122 answers related to the main areas and fields of research in the responding centres (clin. 42, univ. 45, lab. 35). Figure 1 shows the number of responses indicating high or relatively high activity in a specific field.

Research in genetic and pathophysiology is the most active field (108/122, Fig.1a), with a strong predominance of genetics (gene identification 95/122, Fig. 1b) and genomics (high throughput genomics 80/122, Fig. 1b). These activities rely on sampling and data collection (biobanking 69/122, database for omics 67/122, omics technologies 63/122, Fig. 1b) and benefit from shared platforms (for biobanking 64/122, for genomic and proteomic 66/122, Fig. 1f), and to local bioinformatics facilities (60/122, Fig. 1b) rather than shared bioinformatics platforms (36/122, Fig. 1f). Activities in imaging and microscopy (64/122, Fig. 1b) and search for new animal models (63/122, Fig. 1b) indicate interest for research in pathophysiology in about one-half of the responding teams.

The second most active field of activity is therapeutic research (94/122, Fig. 1a), with a slight predominance of investigational studies (81/122, Fig. 1c) over therapeutic phase I/II studies (65/122, Fig. 1c), or phase III trials sponsored by industry (69/122, Fig. 1c). The specific fields of research on transplantation (68/122, Fig.1c), medical devices (50/122, Fig. 1c), or surgical procedures (53/122, Fig. 1c) are each dealt with in about one-half of the responding teams. Few among the responding teams are concerned by research in bio-production (18/122, Fig.1c; GMP platform 35/122, Fig.1h).

Epidemiology is an active field of research in the MetabERN network (79/122, Fig.1a), with marked interest for all aspects of the discipline relevant to rare diseases. The constitution of registries (97/122, Fig.1d) and cohorts (80/122, Fig.1d) benefits from dedicated platforms (registries 76/122, long-term cohorts 80/122, Fig. 1h). They are used for the definition of biomarkers (96/122, Fig.1d) or other methods for diagnosis and prognosis (85/122, Fig.1d), as well as to identify new (78/122, Fig.1d) and/or ultra rare (79/122, Fig.1d) diseases.

Preclinical research in in vitro or in animal models with the purpose of potential therapeutic applications is performed in many of the responding teams (72/122, Fig.1a), although each thematic issue concerns a minority of the teams. They include investigations aimed at defining therapeutic targets (50/122, Fig.1g), therapeutic methods (biotechnology research 50/122, Fig.1g), or producing proof of concept (50/122, Fig.1g). The less active fields concern research on therapeutic chemicals (search for activity, drug screening, toxicology: ≤40/122, Fig. 1e and Fig. 1h).

A large proportion of the responding teams is interested in various aspects of research on health systems (60/122, Fig.1a). These include the validation of methods for diagnostic screening (87/122, Fig.1e), or the elaboration of research policies (guidelines, national plans, health care resources, 85/122, Fig.1e). However, only a minority of the responding teams declared activity in cost analysis studies, health technology assessment, or e-health (45/122, 39/122, 53/122, respectively, Fig. 1e).

The less active research activity domain of the responding teams concerns human and social sciences (37/122, Fig.1a). Activity is low in all aspects, and especially marginal for research aimed at investigating the patient’s quality of life and its determinants (society and rare diseases 41/122, methods to support quality of life 49/122, Fig.1f). Slightly more activity was declared in the fields of care practices, patient awareness and health care policies and innovation (61/122, 64/122, 53/122 and 56/122, respectively, Fig.1f).

### Collaboration with international research infrastructures and organizations

The survey comprised questions referring to knowledge about the existence of various international research organizations and collaboration with these organizations. We received 106 answers (clin. 34, univ. 42, lab. 30, Table 1).

**Table 1:** Numbers of positive responses out of 106 received responses to the questions « do you know » and « do you collaborate with» the following organizations. Full names and/or field of activity of the listed organizations are given in the text

		Number of positive answers	
	Organization	Known	Collaboration
ESFRI Infrastructures	BBMRI	55	14
	EATRIS	33	4
	ECRIN	51	8
	ELIXIR	37	2
	OPENSCREEN	23	0
	EUROBIOIMAGING	29	1
	INFRAFRONTIER	15	0
	ISBE	24	1
	MIRRI	13	1
Registries	E---HOD (homocystinurias, methylation)	66	39
	EIMD	65	37
	Euroglycanet	54	10
	ICG Gaucher reg.	58	18
	Pompe	43	21
	MPS I reg.	53	43
	International PMSI reg.	62	19
	Hunter Outcome survey	58	39
	International Morquio reg.	55	20
	lInternationa Niemann---Pick reg.	61	20
	Fabry Outcome survey	60	30
	International Fabry reg.	66	20
	Galactosemia patient reg.	53	29
International organisations	EURORDIS	87	27
	ISNS	53	15
	SSIEM	93	71
	SIMD	55	15
	ERNDIM(QI)	75	40
	IRDIRC	61	18

The first set of questions concerned collaborations with the international research infrastructures supported by the European Scientific Forum on Research Infrastructures (ESFRI) [6], which proposes services for biomedical research. Services concern biobanking (BBMRI), translational research (EATRIS), clinical research (ECRIN), bioinformatics (ELIXIR), drug screening (OPENSCREEN), imaging (EUROBIO-IMAGING), transgenic animals repository (INFRAFRONTIERS), system biology (ISBE), and a micro-organisms repository (MIRRI). Answers showed comparable scores for the clinical, university and laboratory groups, which indicated that a large majority of the responding centres do not know about the existence of these infrastructures, and even when they know them, they very rarely collaborate with them (Table 1). An additional question concerning 18 national biobanking infrastructures (list in Additional file 1) revealed that in the best case, 47 of the 106 respondents knew about the service and 8 collaborated with it. The large majority of the others national biobanks were not known.

In contrast, responding teams are well aware of the existence of patient registries, even beyond their specific field of interest (Table 1), and actively participate in the constitution of these registries. Clinical teams, university teams, and laboratory teams expressed equal level of interest.

Teams were also asked whether they are aware of the existence of international scientific organizations relevant to rare diseases and participate to their activities (Table 1). Questions concerned EURORDIS (a non-governmental patient-driven alliance of patient organizations), ISNS (the International Society for Neonatal Screening), SSIEM (the Society for the Study of Inborn Errors of Metabolism), SIMD (the Society for Inherited Metabolic Diseases), ERNDIM (the European Research Network for the Diagnosis of Inherited disorders of the Metabolism), and IRDIRC (the International Rare Disease Research Consortium). All these organizations are known by a majority of the responding centres, and many of them actively participate.

## Discussion

This report presents a descriptive analysis of the information collected through a cross-sectional on-line survey performed in December 2017 by MetabERN. According to the missions attributed to the ERNs by the EU cross-border healthcare directive 2011/24 to reinforce medical research in their thematic field, the survey was conducted to get insights into the research activities and capabilities of the members of the MetabERN network. The target audience comprised all of the 69 centres participating in MetabERN, which were designated by the European Commission (DG Sante) as the reference centres for health care delivery in the field of HMDs in Europe [7]. Three-fourth of the centres (52/69) responded to the survey. These centres are in essence multidisciplinary and reputed as centers of excellence for care, teaching and research in the field of HMDs. The survey therefore provides insight into the centres that offer the best cares, diagnosis, treatments and advices patients with HMDs can find in Europe in the context of the cross-border healthcare directive.

The number of centres participating to the survey and their presumed representativeness as reference centres for HMDs, support the idea that the collected information is relevant to the EU as a whole. However, not all EU countries participate to MetabERN, the number of MetabERN centres largely varies between countries, and some centres sent several responses to the survey (18/154 answers). These facts represent possible bias and limit the representativeness of the study. It is also important to take into account that this study, as a survey, relies on self-declarations. Appropriate interpretation should therefore confront the subjective picture emerging from this study with objective data, such as can be obtained from funding agencies, or institutional or national evaluation bodies.

Questionnaires sent to the centres (shown as Additional file 1) contained multiple choices questions related to the medical research performed by individuals in their respective institutions and countries. Questions referred to the level of research activity and the type of research, with an additional focus on awareness and collaboration with national, European, and international medical research facilities and organizations. Due to the centres’ multidisciplinary teams the questions were adapted to the clinical, university and laboratory teams specific interests.

The high response rate is an indication of the marked interest for research among the respondents. They expressed high motivation and commitment to do research, and indicated that the research conditions of their institution were mostly satisfactory. Respondents were actively contributing to research activities, including the clinicians, who nevertheless considered their clinical workload being a serious barrier to research. One solution to this problem could be increasing rotational positions for clinicians and researchers [8]. The high standards of excellence in research are indicated by the prevalence of previous research experience in a foreign country, funding by competitive grants, publications in international scientific journals, and international collaborations. Most respondents are satisfied with the quality and impact of the research performed in their institution, and with the relevance of the research education programs available in their university. Taken as a whole, these results provide an encouraging picture of the research capacities and activities in the MetabERN centres.

Research in the MetabERN centres is primarily performed in the fields of genetics and pathophysiology. There is strong focus on issues related to diagnosis. Predominant activities are studies of the genetic determinants of diseases, the identification of new disease entities, and the search of biomarkers for diagnosis and prognosis. The constitution and the use of patient registries appear highly instrumental for these purposes [9, 10]. Consistently, responding teams are actively collaborating with national and international patient registries in their field of interest, and participate in the definition of healthcare policies and guidelines, such as the implementation of new diagnostic technologies and the recommendations of the ISNS on issues related to neonatal screening [11, 12]. Interestingly, as shown by their frequent participation to the ERNDIM network, responding teams demonstrate their interest for quality assurance issues [13, 14]. It is however surprising in this context that collaboration with the national and international biobanking platforms was rather infrequent [15]. The marginal interest for bioinformatics platforms is also noticeable, suggesting that these infrastructures are not closely linked with medical teams and/or not easily accessible [16].

Therapeutic research performed in the network essentially consists in clinical research, with only modest activity related to preclinical investigations. This finding emphasizes the positioning of the MetabERN network as a medical research network. Although links with more basic biological research exist, as well as projects involving omics technology, imaging, or animal models, these activities are not central for the participating teams. The questionnaires were not intended to collect detailed information on research activities based on clinical trials, as this topic is treated in a currently on-going study. Nevertheless, responses to the question related to therapeutic research indicated that clinical teams were all participating in clinical trials as a current activity. Trials are most often investigational studies, although participation to phase I/II and phase III clinical trials conducted by industrial companies is also high.

A major finding of the survey is the limited implication of the respondents in human and social sciences studies. Only one third or less of the respondents declared having activity in this field, including research on patient’s quality of life, patient’s awareness, or methods for social support. This result points out that the multidisciplinary constitution of the responding teams is only partial, and that they presumably often do not comprise researchers in the field of human and social sciences. This weakness may have practical consequences with respect to the production of relevant knowledge for social intervention, to the design of strategies aimed at empowering patients and developing collaborations with patient’s advocacy groups, and finally to ensure that patient’s needs are effectively taken into account. Therefore, MetabERN should consider actions to be taken in this field as an absolute priority [17]. In contrast to the focus of EU policies on e-health and health technology assessment, respondents declared modest implication in these fields. As these are important issues for the implementation of the cross-border healthcare directive, it would be worth encouraging teams to pay more attention to these matters.

Another unexpected result of the survey is the low awareness of the respondents regarding services made available by the international medical research infrastructures, which have been set up by ESFRI. As many of the proposed services would be highly instrumental for the clinical research teams, this observation suggests an important lack of sufficient communication and knowledge transfer between the teams involved in ESFRI infrastructure management and the teams involved in medical research.

## Conclusion

The survey points out the value of a transnational network like MetabERN to carry out studies capable of giving a broad and in-depth vision of the current status of medical research in a specific field in Europe. The interpretation of these results and the conclusions to be drawn will be highly valuable for the design of research priorities in the network, and might possibly influence research policies on rare diseases beyond the network [18, 19].

## Methods

The spectrum of HMDs cared for in the MetabERN centres can be specified according to the number and type of patients they managed: AOAs count for 39% of the activity (13,372 patients), LSDs 23% (7641), C-FAOs 16% (5349), NOMS 11% (3848), PM-MD 7% (2414), PDs 3% (994), and CDGs 1% (407). HCPs associated in the network split their working time between various types of activities, which in many cases are part of clinical care: prevention and screening (9% of their time), diagnosis and description of new disease entities (12%), patient management and definition of guidelines and pathways (12%), patient empowerment (10%), counseling (8.5%), dissemination and contact with stakeholders (7%), education and training (10%), research in epidemiology and constitution of registries (12.5%), clinical research (10%), and pre-clinical research (8%).

HCPs acting in the centres (*n* = 1671) form multidisciplinary teams composed of specialized medical doctors, many of them having teaching obligations (*n* = 871, 52%), biochemists/biologists (*n* = 188, 11%), pharmacists (*n* = 34, 2%), nurses, dieticians, physical therapists and psychologists (*n* = 454, 27%), social workers (*n* = 49, 3%), managers, coordinators and secretaries (*n* = 75, 9%).

The survey was performed in December 2017/January 2018, asking the 69 MetabERN centres to complete questionnaires on the online SurveyMonkey platform. Considering the variety of the tasks directly or indirectly related to providing care, teaching, or performing research on HMDs, three different questionnaires were addressed to three different groups of actors in each centre: 1) a questionnaire sent to the clinical department (61 questions) was supposed to reach the persons involved in the various aspects of clinical care; 2) a questionnaire sent to the University (37 questions) was aimed at collecting the views of researchers and teachers; 3) a questionnaires sent to laboratories (37 questions) was intended to reach clinical biologists and pharmacists. Questionnaires included questions common to the three groups and specific questions for each group. In total, 126 different questions were asked. Participants were invited to answer questions with multiple choices. The list of the questions sent to each group and the multiple choice answers are shown as Additional file 1. Questions enquired about personnel involved in research, quality and impact of the research performed, motivation and barriers to do research, research funding, level and type of research activity, thematic priorities, access to and use of local, national and international facilities and infrastructures. The responses indicating “high” or “relatively high”, “very important” or “important” were considered together and the corresponding numbers of responses were added.

## Additional file

Additional file 1: **Table S1.** List of the MetabERN centres and participation to the survey (PDF 1220 kb)

## Abbreviations

AOA: Aminoacid and organic acids related disorders
CDG: Ongenital disorders of glycosylation and disorders of intracellular trafficking
C-FAO: Carbohydrate, fatty acid oxidation and ketone bodies disorders
ERN: European Reference Network
ESFRI: European Scientific Forum on Research Infrastructures
EU: European Union
HCP: Health Care Provider
HMD: Hereditary Metabolic Disease
LSD: Lysosomal Storage Disease
NOMS: Disorders of Neuromodulators and Small Molecules
PD: Peroxisomal and lipid related disorders
PM-MD: Disorders of pyruvate metabolism, Krebs cycle defects, mitochondrial oxidative phosphorylation disorders, disorders of thiamine transport and metabolism
