# Supplementary material for: Research activity and capability in the European reference network MetabERN
Source: Orphanet J Rare Dis. 2019 May 29;14:119. doi: 10.1186/s13023-019-1091-8 (PMC6542047; doi:10.1186/s13023-019-1091-8)
Supplement: Supplementary file 1 — Table S1. List of the MetabERN centres and participation to the survey (PDF 1220 kb) [file 13023_2019_1091_MOESM1_ESM.pdf]

Supplementary material

Table S1: List of the MetabERN centres and participation to the survey

Country	MetabERN centres	Number of answers Clin.	Univ.	Lab.	Total
BE	CHU Liège	1	3	2	6
	Cliniques universitaires St Luc-Univ. catholique Louvain	0	1	0	1
	Ghent University hospital	0	0	0	0
	Metabolic Center ULB-VUB	2	2	2	6
	University Hospital of Antwerp UZA	1	1	1	3
	UZ Leuven	2	0	1	3
BU	University hospital "Alexandrovska"in Sofia	0	0	0	0
CR	University Hospital Center Zagreb	0	0	1	1
CZ	General University Hospital in Prague-GUH	1	1	1	3
DK	Copenhagen University Hospital, Rigshospitalet	1	0	0	1
FR	Hôpitaux Universitaires Paris Ile de France Ouest	0	0	0	0
	Hôpitaux Universitaires Paris Nord Beaujon	1	2	0	3
	Hôpitaux Universitaires Lyon	0	0	0	0
	Hôpitaux Universitaires Paris Nord Antoine-Béclère	1	1	0	2
	Hôpitaux Universitaires Paris Nord Louis-Mourier	1	1	1	3
	Hôpitaux Universitaires Paris Necker-Enfants Malades	0	1	0	1
	Hôpitaux Universitaires Paris Robert-Debré	0	0	1	1
	Hôpitaux Universitaires Lille	1	1	1	3
	Hôpitaux Universitaires Marseille	0	0	0	0
DE	Helios Dr. Horst Schmidt Kliniken	0	0	1	1
	Charité-Universitätsmedizin Berlin	1	1	0	2
	Hannover Medical School	0	0	0	0
	Medical Center University of Freiburg	1	2	2	5
	Muenster University Hospital	0	0	1	1

	Otto-von-Guericke-University Magdeburg	0	0	0	0
	Universitätskinderklinik Giessen	1	2	1	4
	Universitätsklinikum Heidelberg	1	1	1	3
	University Medical Center Hamburg-Eppendorf	1	1	1	3
	University Medical Center Mainz	2	1	1	4
HU	Hungary-University of Debrecen	1	1	1	3
IT	Azienda Sanitaria Universitaria Integrata di Udine	2	1	1	4
	Meyer Children's Hospital Firenze	0	0	0	0
	ASST Monza San Gerardo Hospital	1	2	0	3
	Azienda Ospedaliera Universitaria "Federico II", Napoli	1	1	2	4
	Azienda Ospedaliera Universitaria Integrata di Verona	0	0	0	0
	Ospedale Pediatrico Bambino Gesù, Roma	0	0	0	0
	Giannina Gaslini Institute, Genova	1	0	1	2
	San Paolo Hospital, ASST Santi Paolo e Carlo, Milano	1	1	1	3
	University Hospital of Padova	1	1	0	2
	Azienda Ospedaliero-Universitaria Pisana	0	1	0	1
	Azienda Ospedaliera Universitaria Senese	2	2	2	6
LI	Vilnius University Hospital Santariskiu klinikos	0	0	0	0
NL	Erasmus MC, University Medical Center, Rotterdam	2	1	0	3
	Maastricht University Medical Center	0	0	1	1
	University Medical Center of Groningen	2	1	0	3
	University Medical Center Utrecht (UMCU)	1	1	1	3
	Academic Medical Center Amsterdam	1	0	1	2
NO	Oslo University Hospital	1	2	1	4
	Helse Bergen HF, Haukeland University Hospital	0	0	0	0
PL	Krakow University Hospital	1	1	4	6
PO	Centro Hospitalar Lisboa Norte, EPE	1	1	1	3
	Centro Hospitalar do Porto, EPE (CHP)	1	1	0	2
	Centro Hospitalar do Sao Joao, E.P.E.	1	1	2	4

	Centro Hospitalar e Universitário de Coimbra, E.P.E.	2	1	1	4
	Hospital Senhora da Oliveira – Guimarães	0	0	0	0
SO	University Medical Centre Ljubljana	1	1	1	3
SP	University Clinical Hospital of Santiago de Compostela.	1	0	0	1
	Hospital Sant Joan de Déu, Barcelona	1	2	0	3
	Hospital Universitario Cruces, Vizcaya	1	1	1	3
	Hospital Universitario Vall d'Hebron, Barcelona	1	0	0	1
	Hospital Universitario "12 de Octubre" Madrid	1	2	1	4
SW	Karolinska University Hospital, Solna	2	1	0	3
	Sahlgrenska University Hospital University of Gothenburg	0	1	1	2
UK	Cambridge University Hospitals	0	0	0	0
	Birmingham Children’s Hospital	1	2	1	4
	University Hospitals Bristol, Bristol Children's Hospital	0	0	0	0
	Central Manchester University Hospitals	0	0	0	0
	Great Ormond Street Hospital London	0	0	0	0
	University Hospital of Birmingham	1	0	0	1
		**52**	**52**	**44**	**148**

European Reference Networks

Survey MetabERN for Departments

*1. Can you please type in your contact details

Name

Email Address

Phone Number

*2. Please select the name of your hospital /institute from the drop down menu

3. Please select what is applicable to your situation

- male
- female

4. Name of the higher education institution (in original language)

5. Faculty/ department/ institute (in English)

6. Name of the faculty/ department/ institute (in original language)

7. Type of institution

- Public university
- Private university

- ☐ Public college
- ☐ Private college
- ☐ Research institute

*8. What is the legal status of your institution

- ○ Public
- ○ Private
- ○ mixed

*9. What is your position

- ○ Professor
- ○ Associate Professor
- ○ Lecturer
- ○ Senior Research Fellow
- ○ Postdoc/Research Fellow
- ○ Research Assistant
- ○ Teaching Assistant
- ○ Dean, head of department or other senior administration position
- ○ Other (please specify)

*10. Please select the areas and fields in which your institution is undertaking research activities, please select all the ones of interest:

- [ ] Animal research
- [ ] Basic research
- [ ] Preclinical research
- [ ] Clinical research

---

*11. Please indicate which are, the main research activities performed inside your Institution

	No activity	Low activity	Rather low activity	Rather high activity	High activity
Health Systems Research	○	○	○	○	○
Human and Social Science Research	○	○	○	○	○
Epidemiology/Natural History of diseases	○	○	○	○	○
Research in Genetics and Pathophysiology	○	○	○	○	○
Pre-Clinical Therapeutic Research	○	○	○	○	○
Therapeutic Research	○	○	○	○	○
Research Platforms/Infrastructures	○	○	○	○	○
Development and availability of tools needed to accelerate research on rare diseases	○	○	○	○	○

*12. **Academic** Health Systems Research

	No activity	Low activity	Rather low activity	Rather high activity	High activity
Research policy (e.g., guidelines development; national plans; health care resources)	○	○	○	○	○
Cost analysis (e.g., guidelines implementation)	○	○	○	○	○
New technologies (e.g., New diagnostic tests, population screening programmes, Innovative therapeutics)	○	○	○	○	○
Health-Technology assessment of Orphan Drugs	○	○	○	○	○
e-Health activities	○	○	○	○	○

*13. **Industrial** Health Systems Research

	No activity	Low activity	Rather low activity	Rather high activity	High activity
Research policy (e.g., guidelines development; national plans; health care resources)	○	○	○	○	○
Cost analysis (e.g., guidelines implementation)	○	○	○	○	○
New technologies (e.g., New diagnostic tests, population screening programmes, Innovative therapeutics)	○	○	○	○	○
Health-Technology assessment of Orphan Drugs	○	○	○	○	○
e-Health activities	○	○	○	○	○

*14. Human and social science research

	No activity	Low activity	Rather low activity	Rather high activity	High activity
Society and RD (e.g., social perception, accessibility to care,…)	◌	◌	◌	◌	◌
RD, Research and Innovation (e.g., public/private cooperation, role of patients org,….)	◌	◌	◌	◌	◌
Care practices (self- management, health education policy,…)	◌	◌	◌	◌	◌
Patients awareness (e.g., understanding genetic data, importance of animal models,…)	◌	◌	◌	◌	◌
Support for caregivers	◌	◌	◌	◌	◌
Public Heath and RD (e.g., health care policies, prospective public policies,…)	◌	◌	◌	◌	◌
Methods for social support to improve quality of life	◌	◌	◌	◌	◌
Services accessibility	◌	◌	◌	◌	◌
Orphan Drugs availability	◌	◌	◌	◌	◌
Methods to assess QoL	◌	◌	◌	◌	◌

*15. Epidemiology & Natural History of diseases

	No activity	Low activity	Rather low activity	Rather high activity	High activity
Registries	◌	◌	◌	◌	◌
Databases/data management systems for epidemiology studies	◌	◌	◌	◌	◌
Cohorts	◌	◌	◌	◌	◌
Biomarkers to assess diagnosis, prognosis and severity of diseases	◌	◌	◌	◌	◌
Diagnosis/prognosis methods (e.g., other than biomarkers)	◌	◌	◌	◌	◌
Identification of new entities	◌	◌	◌	◌	◌
Networks for ultra rare diseases	◌	◌	◌	◌	◌

*16. Research in genetics and physiopathology

	No activity	Low activity	Rather low activity	Rather high activity	High activity
Gene identification and Molecular Mechanisms	◌	◌	◌	◌	◌
New database method developments to be applied to omics research	◌	◌	◌	◌	◌
Biobanking research	◌	◌	◌	◌	◌
High throughput genomics/sequencing	◌	◌	◌	◌	◌
New bioinformatic methods and developments	◌	◌	◌	◌	◌
New technologies (e.g., atomic microscopy…)	◌	◌	◌	◌	◌

	No activity	Low activity	Rather low activity	Rather high activity	High activity
Omics technology (e.g., proteo, metabolo..)	○	○	○	○	○
Development of new animal models	○	○	○	○	○
New Imaging/Microscopy methods	○	○	○	○	○
Development of new therapeutic tools	○	○	○	○	○

*17. Pre-clinical therapeutic research

	No activity	Low activity	Rather low activity	Rather high activity	High activity
Proof-of-concept	○	○	○	○	○
Drug libraries	○	○	○	○	○
Search for active chemical compounds	○	○	○	○	○
High throughput drug screening	○	○	○	○	○
Therapeutic target identification/validation (e.g.,. stem cells, systems biology)	○	○	○	○	○
Cell therapy research (e.g., viral vectors…)	○	○	○	○	○
Innovative biotechnological research (e.g., monoclonal antibodies, cell and gene therapy, enzyme therapy…)	○	○	○	○	○
Drug toxicology/pharmacology	○	○	○	○	○

*18. Therapeutic research

	No activity	Low activity	Rather low activity	Rather high activity	High activity
Drug development	◌	◌	◌	◌	◌
Phase I/II Clinical Trials	◌	◌	◌	◌	◌
Bioproduction centres	◌	◌	◌	◌	◌
Epidemiological/clinical/ therapeutical investigations	◌	◌	◌	◌	◌
Medical devices	◌	◌	◌	◌	◌
Assesment of surgery procedures	◌	◌	◌	◌	◌
Transplants	◌	◌	◌	◌	◌
Pharma/academic partnerships	◌	◌	◌	◌	◌

*19. Platforms / Infrastructures development

	No activity	Low activity	Rather low activity	Rather high activity	High activity
Bioinformatics	◌	◌	◌	◌	◌
Pharmacogenomic diagnostic assays	◌	◌	◌	◌	◌
Population Registries	◌	◌	◌	◌	◌
Genetically-modified animal model repositories	◌	◌	◌	◌	◌

	No activity	Low activity	Rather low activity	Rather high activity	High activity
GMP platforms	○	○	○	○	○
Biobanking	○	○	○	○	○
Genomic and proteomic platforms	○	○	○	○	○
Long-term cohort of patients studies	○	○	○	○	○
High throughput drug screening	○	○	○	○	○

*20. Human resources

	No activity	Low activity	Rather low activity	Rather high activity	High activity
Rotational positions for clinicians	○	○	○	○	○
Rotational positions for researchers	○	○	○	○	○
Specific training in RD	○	○	○	○	○
Training in bioinformatics	○	○	○	○	○

*21. Development and availability of tools needed to accelerate research on rare disease.

	No activity	Low activity	Rather low activity	Rather high activity	High activity
Network development	◌	◌	◌	◌	◌
Creation of transnational research consortiums	◌	◌	◌	◌	◌
Transnational calls for projects funding	◌	◌	◌	◌	◌
Opening of national research programmes to international collaboration	◌	◌	◌	◌	◌
Transnational research infrastructures	◌	◌	◌	◌	◌
Development of an inventory/directory of databases of experts	◌	◌	◌	◌	◌
Transnational collection of funded RD research projects	◌	◌	◌	◌	◌

*22. What influences and governs research at your institution?

	Strongly agree	Agree to some extent	Disagree to some extent	Strongly disagree	No information available	Not applicable
Ministry/ national research agency has a strong say	◌	◌	◌	◌	◌	◌
Central university level mostly decides	◌	◌	◌	◌	◌	◌
Faculties/ departments/ institutes decide very much on what they do	◌	◌	◌	◌	◌	◌
Individual professors/ Researchers do very much what they want	◌	◌	◌	◌	◌	◌
In my own position, I can influence research agendas at my faculty/department/ institute	◌	◌	◌	◌	◌	◌

*23. What is the main impact of the research activities undertaken at your faculty/ department / institute?

	high impact	impact to some extent	low to none impact	do not know	Not applicable
Contribute to international research	○	○	○	○	○
Contribute to national research	○	○	○	○	○
Support of local/ regional innovation of development	○	○	○	○	○
Informing teaching, to be sure that teaching is based on up-to- date research	○	○	○	○	○
Other	○	○	○	○	○

*24. What is the approximate number of staff in research and teaching at your faculty/department/ institute?

	total number
Full Professors	[▾] Full Professors total number menu
Nurses with responsibilities for research only	[▾] Nurses with responsibilities for research only total number menu

	total number
Physicians with responsibilities for research only	Physicians with responsibilities for research only total number menu
PhDs with responsibilities for research only	PhDs with responsibilities for research only total number menu
Nurses with responsibilities for teaching only	Nurses with responsibilities for teaching only total number menu
Physicians with responsibilities for teaching only	Physicians with responsibilities for teaching only total number menu
PhD with responsibilities for teaching only	PhD with responsibilities for teaching only total number menu
Nurses with responsibilities for teaching and researching	Nurses with responsibilities for teaching and researching total number menu
Physicians with responsibilities for teaching and researching	Physicians with responsibilities for teaching and researching total number menu
PhD with responsibilities for teaching and researching	PhD with responsibilities for teaching and researching total number menu

25. What is the approximate total working days of your staff for teaching and/or research ? (If you don't have Prof 2,3 etc and PhD 4,5, etc, you just fill in 0 in all columns, if you are able to fill in how many hours they teach and do research you can fill in 0 at the last column. If you are not able to specify between teaching and researching you fill in 0 in column 1 and 2 and you estimate the total working days in column 3)

	Days of teaching only	Days of research only	Always different but works for
Professor 1	Professor 1 Days of teaching only menu	Professor 1 Days of research only menu	Professor 1 Always different but works for menu
Professor 2	Professor 2 Days of teaching only menu	Professor 2 Days of research only menu	Professor 2 Always different but works for menu

	Days of teaching only	Days of research only	Always different but works for
Professor 3	Professor 3 Days of teaching only menu	Professor 3 Days of research only menu	Professor 3 Always different but works for menu
Professor 4	Professor 4 Days of teaching only menu	Professor 4 Days of research only menu	Professor 4 Always different but works for menu
Professor 5	Professor 5 Days of teaching only menu	Professor 5 Days of research only menu	Professor 5 Always different but works for menu
PhD 1	PhD 1 Days of teaching only menu	PhD 1 Days of research only menu	PhD 1 Always different but works for menu
PhD 2	PhD 2 Days of teaching only menu	PhD 2 Days of research only menu	PhD 2 Always different but works for menu
PhD 3	PhD 3 Days of teaching only	PhD 3 Days of research only menu	PhD 3 Always different but works for menu

	Days of teaching only menu	Days of research only	Always different but works for
Phd 4	Phd 4 Days of teaching only menu	Phd 4 Days of research only menu	Phd 4 Always different but works for menu
PhD 5	PhD 5 Days of teaching only menu	PhD 5 Days of research only menu	PhD 5 Always different but works for menu
PhD 6	PhD 6 Days of teaching only menu	PhD 6 Days of research only menu	PhD 6 Always different but works for menu
Physician 1	Physician 1 Days of teaching only menu	Physician 1 Days of research only menu	Physician 1 Always different but works for menu
Physician 2	Physician 2 Days of teaching only menu	Physician 2 Days of research only menu	Physician 2 Always different but works for menu
Physician 3	Physician 3 Days of teaching only menu	Physician 3 Days of research only menu	Physician 3 Always different but works for menu
Physician 4	Physician 4 Days of teaching only menu	Physician 4 Days of research only menu	Physician 4 Always different but works for menu
Physician 5	Physician 5 Days of teaching only	Physician 5 Days of research only menu	Physician 5 Always different but works for menu

	Days of teaching only menu	Days of research only	Always different but works for
Physician 6	Physician 6 Days of teaching only menu	Physician 6 Days of research only menu	Physician 6 Always different but works for menu
Physician 7	Physician 7 Days of teaching only menu	Physician 7 Days of research only menu	Physician 7 Always different but works for menu
Nurse 1	Nurse 1 Days of teaching only menu	Nurse 1 Days of research only menu	Nurse 1 Always different but works for menu
Nurse 2	Nurse 2 Days of teaching only menu	Nurse 2 Days of research only menu	Nurse 2 Always different but works for menu
Nurse 3	Nurse 3 Days of teaching only menu	Nurse 3 Days of research only menu	Nurse 3 Always different but works for menu
Nurse 4	Nurse 4 Days of teaching only menu	Nurse 4 Days of research only menu	Nurse 4 Always different but works for menu
Nurse 5	Nurse 5 Days of teaching only menu	Nurse 5 Days of research only menu	Nurse 5 Always different but works for menu
Nurse 6	Nurse 6 Days of teaching only	Nurse 6 Days of research only menu	Nurse 6 Always different but works for menu

	Days of teaching only menu	Days of research only	Always different but works for
Nurse 7	Nurse 7 Days of teaching only menu	Nurse 7 Days of research only menu	Nurse 7 Always different but works for menu
Nurse 8	Nurse 8 Days of teaching only menu	Nurse 8 Days of research only menu	Nurse 8 Always different but works for menu

Do you have more staff? Can you please specify.

*26. Do colleagues at your faculty/ department/ institute have access to the following facilities and infrastructures and rate their quality:

	International standard	Good national standard	Insufficient	Do not know	Not applicable
Research support services / unit	○	○	○	○	○
Research laboratories	○	○	○	○	○
Teaching labs (if different from the research Labs)	○	○	○	○	○
Large research equipment that requires technicians	○	○	○	○	○
Scientific databases (e.g. ISI Web of Knowledge)	○	○	○	○	○

	International standard	Good national standard	Insufficient	Do not know	Not applicable
Knowledge Transfer Office	◌	◌	◌	◌	◌
Scientific Park	◌	◌	◌	◌	◌
Research ethics committee	◌	◌	◌	◌	◌
Libraries	◌	◌	◌	◌	◌
Research publication repositories	◌	◌	◌	◌	◌
Computer equipment	◌	◌	◌	◌	◌
IT Services	◌	◌	◌	◌	◌
Accommodation for visiting fellows/researchers	◌	◌	◌	◌	◌
Buildings/premi ses in general	◌	◌	◌	◌	◌
Communication infrastructure (phone, internet etc.)	◌	◌	◌	◌	◌
Data Sharing Platform	◌	◌	◌	◌	◌

*27. Do your faculty/ department/ institute members engage in the following activities?

	Common practice	Possible but rarely used/done	Not practiced	Not possible, due to regulations/ laws	Do not know	Not applicable
Collaborative research with international companies	◌	◌	◌	◌	◌	◌

	Common practice	Possible but rarely used/done	Not practiced	Not possible, due to regulations/ laws	Do not know	Not applicable
Contract research for international companies	○	○	○	○	○	○
Collaborative research with domestic companies	○	○	○	○	○	○
Contract research for domestic companies	○	○	○	○	○	○
Working in industry (contract or part-time)	○	○	○	○	○	○
Spin-off creation	○	○	○	○	○	○
Patent activity	○	○	○	○	○	○

*28. How would you rate the quality of the following at your faculty/ department/ institute?

	International standard, or close to it	Good national standard	Insufficient	Do not know	Not applicable
Research Excellence	○	○	○	○	○
Attractive institutional environment	○	○	○	○	○
Industry contacts and collaboration	○	○	○	○	○
International networking	○	○	○	○	○

	International standard, or close to it	Good national standard	Insufficient	Do not know	Not applicable
Quality Assurance	○	○	○	○	○

*29. Do you award doctorates

- ○ Yes
- ○ No

*30. How frequently are the following?

	Common practice	Possibly but rarely used	Not practiced	Not possible due to regulations/laws
Doctoral thesis can be written in English	○	○	○	○
PhD courses are taught in English	○	○	○	○
Transferable skills training is provided	○	○	○	○
Interdisciplinary research is encouraged	○	○	○	○
International mobility for doctoral candidates (to attend conferences, access libraries and labs abroad)	○	○	○	○
International exchange of doctoral candidates	○	○	○	○
International joint doctoral Programmes	○	○	○	○
Programmes in collaboration with industry	○	○	○	○

	Common practice	Possibly but rarely used	Not practiced	Not possible due to regulations/laws
Support units for doctoral studies	○	○	○	○
Research study rooms for candidates	○	○	○	○
Provision of accommodation for doctoral candidates	○	○	○	○

Other (please specify)

*31. Have you participated in any clinical trials before?

- ○ Yes
- ○ No

*32. What was your role in the latest clinical trial that you have participated in?

- ○ Not participated in clinical trial
- ○ Principal investigator
- ○ Co-investigator
- ○ Sub-investigator

*33. What do you think are the benefits of conducting clinical trials?

	Strongly agree	Agree	Agree to some extent	Disagree
Doctors may help patients with new treatments	◌	◌	◌	◌
Doctors can contribute to medical progress	◌	◌	◌	◌
Doctors can obtain a wider and deeper understanding of the disease	◌	◌	◌	◌
Doctors can obtain research grants or other rewards	◌	◌	◌	◌
Doctors can enhance their reputation in society or in the hospital	◌	◌	◌	◌
Doctors can obtain credits to be a board certified doctor	◌	◌	◌	◌
Doctors can write papers about the clinical trials	◌	◌	◌	◌
The clinical trial will have benefits for patients	◌	◌	◌	◌
Conducting clinical trial is just a waste of time and energy	◌	◌	◌	◌

*34. What are the major problems in conducting clinical trials?

	A serieus problem	A problem	A small problem	No problem
Lack of time for clinicians	◌	◌	◌	◌
Shortage of clinical research coordinators/	◌	◌	◌	◌
research nurses	◌	◌	◌	◌
Difficulties with data management and/or	◌	◌	◌	◌

	A serieus problem	A problem	A small problem	No problem
statistical analysis	◯	◯	◯	◯
Insufficiency of infrastructure	◯	◯	◯	◯
Lack of systemic support from the hospital	◯	◯	◯	◯
Difficulties in communication (e.g	◯	◯	◯	◯
ethical committee,	◯	◯	◯	◯
Enrollment of trial participants	◯	◯	◯	◯
Obtaining of informed consent	◯	◯	◯	◯
Lack of communication with trial sponsor	◯	◯	◯	◯
Funding	◯	◯	◯	◯
Signing of the contract between the company and the hospital administration	◯	◯	◯	◯

Other difficulties to be listed

*35. Have you ever participated in a multinational clinical trial?

◯ Yes

◯ No

*36. What are in your opinion the major obstacles in participation in a multinational clinical trial?[CB1]

[CB1]The following two questions will be linked to the answer to question 7 (yes or no) and we could separately analyse the answers form the yes and no group

	A serieus problem	A problem	A small problem	No problem
Investigator qualifications	○	○	○	○
Quality of clinical trials	○	○	○	○
Infrastructure of clinical trials	○	○	○	○
Difference of the medical system	○	○	○	○
Regulation process and legal system	○	○	○	○
Language barriers	○	○	○	○
Cost-effectiveness	○	○	○	○

Other difficulties to be listed

*37. What do you think is important for overcoming the obstacles for the development of clinical trials?

	Very important	Important	Partially important	Not important
Reform of the system and regulatory rules	○	○	○	○
Financial support to improve the quality of clinical trials	○	○	○	○
Training specialized people in clinical trials	○	○	○	○

	Very important	Important	Partially important	Not important
Establishment of clinical trial centers	◌	◌	◌	◌
Good training programs for clinical trials	◌	◌	◌	◌
Public relations to citizens	◌	◌	◌	◌

*38. Are you interested in conducting clinical trials?

- ◌ Yes
- ◌ No

*39. Who decides on how this budget is allocated to the faculties/ departments?

- ◌ Institutional leadership (Dean, president, director)
- ◌ A central body of the institution (academic senate or similar)
- ◌ Ministry/ national agency

*40. How would you generally describe facilities and infrastructure at your institution

	International standard or close to it	Good national standard	Insufficient	Do not know	Not applicable
Research support services / unit	◌	◌	◌	◌	◌

	International standard or close to it	Good national standard	Insufficient	Do not know	Not applicable
Research laboratories					
Teaching labs (if different from research Labs)					
Large research equipment that requires technicians					
Scientific databases (e.g. ISI Web of Knowledge)					
Knowledge Transfer Office					
Science Park					
Research ethics committee					
Libraries					
Research publication repositories					
Computer equipment					
IT Services					
Accommodation for visiting scholars					
Buildings/premi ses in general					
Power supply/ electricity					
Communication infrastructure (phone, internet etc.)					

*41. Does your institution have, or engages in, any of the following

	Yes very strong	Yes occasionally / some	No	Do not know	Not applicable
Spin-offs	○	○	○	○	○
Research collaboration with industry	○	○	○	○	○
International research collaboration	○	○	○	○	○
Research collaboration with public/ state offices and agencies	○	○	○	○	○
Patents	○	○	○	○	○

*42. Has your institution been involved in applications for research projects (regardless whether successful or not)?

	Frequently as an applicant	Frequently as a partner	Occasionally as an applicant	Occasionally as partner	No involvement of application
National, involving colleagues from another institution	○	○	○	○	○
Horizon 2020 / FP7	○	○	○	○	○
Other European project	○	○	○	○	○
Other International project	○	○	○	○	○

*43. When have you been last research active as a health professional?

(Research activity is defined here as being involved in the design or implementation of a research project either as a researcher or a supervisor)

- Within the last 2 years
- More than 2 years ago
- Never been research active as a health professional

*44. Have you published any of the following over the last 3 years 2014-2017?

	Yes mostly with single authorship	Yes mostly co-authored	Only supervising	No
ISI journal articles				
Other journal articles (non-ISI)				
Papers presented at international conferences				
Papers presented at national conferences				
PhD. Thesis				
Books				
Book chapters				
Reports				

*45. If co-authored, who do you write with more often?

	Colleagues from my institution	Colleagues from other institutions	European Colleagues	Intercontinental Colleagues	No co-authoring
ISI journal articles	○	○	○	○	○
Other journal articles (non-ISI)	○	○	○	○	○
Papers presented at international conferences	○	○	○	○	○
Papers presented at national conferences	○	○	○	○	○
PhD. Thesis	○	○	○	○	○
Books	○	○	○	○	○
Book chapters	○	○	○	○	○
Reports	○	○	○	○	○

` *46. What factors had motivated you to be research active?

	Did not motivate me	Yes some motivation	very strong motivation	N/A
Personal motivation', '	○	○	○	○
Completing an academic degree'	○	○	○	○
Professional development	○	○	○	○
Career advancement	○	○	○	○

	Did not motivate me	Yes some motivation	very strong motivation	N/A
Part of the professional role	○	○	○	○
For publication	○	○	○	○
To gain more expertise	○	○	○	○

*47. If not active in research define the potential barriers to research engagement: If active in research: Is there anything you can point out that prevents or discourages you from engaging in research.

- ☐ Not enough time available to engage in research
- ☐ Clinical workload pressures
- ☐ Lack of support/encouragement
- ☐ Lack of funding
- ☐ Lack of available resources for research
- ☐ Difficulties gaining ethical approval
- ☐ Research not valued in organisation
- ☐ Weaknesses in research skills
- ☐ Personal circumstances (e.g., family commitments limiting time for research)
- ☐ Lack of research opportunities
- ☐ Lack of supervision/mentorship
- ☐ Difficulties collecting or gaining access to data
- ☐ There is no reason why people should be limited or not engaged in research

*48. What are the main problems that researchers face at your institution

	Strongly agree	Agree to some extent	Disagree to some extend	Strongly disagree	Do not know	N/A
There is no or insufficient research funding	○	○	○	○	○	○
There are no or insufficient research facilities	○	○	○	○	○	○
Some research facilities can only be accessed by some researchers	○	○	○	○	○	○
There is no or insufficient administrative capacity to prepare research proposals, manage research projects etc.)	○	○	○	○	○	○
Most researchers have no international research contacts	○	○	○	○	○	○
Most researchers are not internationally mobile	○	○	○	○	○	○
It is not possible to hire international researchers	○	○	○	○	○	○
Most researchers have to teach, and cannot put enough focus on their research	○	○	○	○	○	○
Most researchers have other jobs on the side and cannot put enough focus on their research	○	○	○	○	○	○

*49. What do you think are the reasons why people in your Institution are not, or limited engaged in research?

- ☐ Not enough time available to engage in research
- ☐ Clinical workload pressures
- ☐ Lack of support/encouragement
- ☐ Lack of funding
- ☐ Lack of available resources for research
- ☐ Difficulties gaining ethical approval
- ☐ Research not valued in organisation
- ☐ Weaknesses in research skills
- ☐ Personal circumstances (e.g., family commitments limiting time for research)
- ☐ Lack of research opportunities
- ☐ Lack of supervision/mentorship
- ☐ Difficulties collecting or gaining access to data
- ☐ There is no reason why people should be limited or not engaged in research

*50. What do you see as the main problems for research in your **country**?

	strongly agree	agree to some extent	neither agree nor disagree	disagree	strongly disagree	Do not know
Research is not given sufficiently high priority by the state	○	○	○	○	○	○
There is no national strategy, which would provide a clear framework	○	○	○	○	○	○
There is no or insufficient research funding	○	○	○	○	○	○

	strongly agree	agree to some extent	neither agree nor disagree	disagree	strongly disagree	Do not know
There are no or insufficient research facilities	○		○	○	○	○
Research is too much concentrated in a few institutions	○	○	○	○	○	○
Higher education institutions are focusing too much on teaching, and neglect research	○	○	○	○	○	○
Research is not sufficiently applied, and ignores the needs of the industry and society	○	○	○	○	○	○
There is too much focus on applied research	○	○	○	○	○	○
Interdisciplinary research is not sufficiently encouraged	○	○	○	○	○	○
The best researchers are leaving the country, which is a problem	○	○	○	○		○
The best researchers are leaving the country, but they still continue to Contribute to national research	○	○	○	○	○	○
Young researchers are leaving the country	○	○	○	○	○	○
Young talented people look for careers outside of the research and higher Education sectors	○	○	○	○	○	○

*51. Of the changes listed in the table below, indicate how effective you believe they would be for increasing the level of research activity in your Institution

	Would have no effect	Would be somewhat effective	Would be greatly effective
Increased protected working time for research	◌	◌	◌
Higher levels of ongoing research training	◌	◌	◌
Recruiters in the health service giving more value to research experience when selecting candidates for clinical positions	◌	◌	◌
A more simplified and efficient ethics application process	◌	◌	◌
Greater levels of mentorship and support when conducting research	◌	◌	◌
More opportunities to advance one’s research qualifications (e.g. post-doctoral fellowships)	◌	◌	◌
More funded research posts	◌	◌	◌
More opportunities for collaboration with other clinical or academic researchers	◌	◌	◌

*52. About research Infrastructure, what is applicable for you?

	Don't know it	Know it	Collaborate with it
BBMRI Biobanking and Biomolecular Resources Research Infrastructure	◌	◌	◌
C-ERIC Central-European Research Infrastructures Consortium	◌	◌	◌
CLARIN Common Language Resources and Technology Infrastructure	◌	◌	◌
EATRIS European Advanced Translational Research Infrastructure in Medicine	◌	◌	◌
ECRIN European Clinical Research Infrastructure Network	◌	◌	◌

	Don't know it	Know it	Collaborate with it
ELIXIR The European Life-Science Infrastructure for Biological Information	○	○	○
ERA European Research Area	○	○	○
ERIC European Research Infrastructure Consortium	○	○	○
ESFRI European Strategy Forum on Research Infrastructure	○	○	○
EU-OPENSCREEN European Infrastructure of Open Screening Platforms for Chemical Biology	○	○	○
Euro-BioImaging European Research Infrastructure for Imaging Technologies in Biological and Biomedical Sciences	○	○	○
INFRAFRONTIER European Research Infrastructure for the generation, phenotyping, archiving and distribution of mouse	○	○	○
ISBE Infrastructure for Systems Biology Europe	○	○	○
MIRRI Microbial Resource Research Infrastructure disease models	○	○	○
NRRI Research Infrastructure Roadmap	○	○	○
PRACE Partnership for Advanced Computing in Europe	○	○	○
RI Research infrastructure(s)	○	○	○

*53. About biobanks, what is applicable for you?

	Don't know it	Know it	Collaborate with it
Basque Biobank	○	○	○
Biobanco.pt (Portuguese National Consortium of Biobanks)	○	○	○

	Don't know it	Know it	Collaborate with it
Biobank Inherited Metabolic Disorders (blood/urine/liquor/fibroblasts)	○	○	○
Biobank of the Estonian genome center	○	○	○
BioNER: Biobank of the Institute of Rare Diseases Research/Institute of Health Carlos III	○	○	○
Brain-net – brain tissue bank Aachen	○	○	○
Brain-Net – brain tissue bank Munster (pediatric neuropathology)	○	○	○
Brain-Net Germany : National network of brain banks in Germany	○	○	○
Cell line and DNA Biobank from patients affected by genetic diseases	○	○	○
CIBERER Biobank (Biobank of rare diseases)	○	○	○
DNA biobank of the Department of Molecular Genetics and Diagnostics, National Institute of Environmental Health (NIEH) (EuroBioBank partner)	○	○	○
Genetic alliance Registry and Biobank	○	○	○
EuroBioBank European Network of DNA, cell and tissue banks for Rare Diseases	○	○	○
Human DNA and cell biobank of Genethon – genetic diseases, mainly neuromuscular diseases – part of the EuroBioBank network	○	○	○
Human tissue biobank of Neuromuscular diseases (EuroBioBank partner)	○	○	○
Neuromuscular Tissue Bank (NMTB) (EuroBioBank partner)	○	○	○
Telethon Network of Genetic Biobanks	○	○	○
UK Biobank	○	○	○

*54. About registry and database, what is applicable for you?

	Don't know it	Know it	Collaborate with it
(NCARDRS) National Congenital Anomaly and Rare Disease Registration Service	◌	◌	◌
Austrian Country Node of the Human Variome Project (HVP)	◌	◌	◌
Austrian registry for inborn errors of metabolism	◌	◌	◌
Belgian Neuromuscular Disease Registry	◌	◌	◌
Belgian patient database for Wilson disease – contributes to the EuroWilson registry (terminated)	◌	◌	◌
Biobank Inherited Metabolic Disorders (blood/urine/liquor/fibroblasts)	◌	◌	◌
Canadian Neuromuscular Disease Registry – part of the TREAT-NMD network	◌	◌	◌
DDRMD – Dutch Diagnosis Registration Metabolic Diseases	◌	◌	◌
Dutch Neuromuscular Database CRAMP: Computer Registry of All Myopathies and Polyneuropathies	◌	◌	◌
Dutch patient registry for Fabry disease	◌	◌	◌
Dutch patient registry for Gaucher disease	◌	◌	◌
Dutch patient registry for Niemann-Pick Disease Type B	◌	◌	◌
Dutch patient registry for Niemann-Pick Disease Type C	◌	◌	◌
ECARUCA: cytogenetic and clinical database on rare chromosomal disorders	◌	◌	◌
E-HOD European Network and Registry for Homocystinurias and Methylation Defects	◌	◌	◌
EIMD: European registry and network for intoxication type metabolic diseases	◌	◌	◌
English alpha-1 antitrypsin deficiency registry – contributes to the Alpha One International Registry (AIR)	◌	◌	◌
English cystinosis registry	◌	◌	◌

	Don't know it	Know it	Collaborate with it
English hyperoxaluria registry	◌	◌	◌
English phenylketonuria registry	◌	◌	◌
EPNET: European porphyria Network	◌	◌	◌
E-RARE Consortium	◌	◌	◌
ESNEK – Rare paediatric neurological disease registry Germany	◌	◌	◌
EUNEFRON: registry of the European network for the study of orphan nephropathies	◌	◌	◌
EUROCAT European Surveillance on congenital anomalies	◌	◌	◌
EUROGLYCANET – International patient registry and cohort for congenital disorders of glycosylation	◌	◌	◌
European cystinosis cohort	◌	◌	◌
European Porphyria Registry (EPR)	◌	◌	◌
Fabry Disease Registry –DE	◌	◌	◌
FOS : Fabry Outcome Survey	◌	◌	◌
French certified registry of glycogen storage disease type 2	◌	◌	◌
French certified registry of patients affected by Gaucher disease	◌	◌	◌
French cystinosis registry	◌	◌	◌
French registry of neuromuscular diseases from reference centres	◌	◌	◌
Galactosemia Patient Registry – part of the Galactosemia Network	◌	◌	◌
Genetic alliance Registry and Biobank	◌	◌	◌
German mucopolysaccharidosis patient registry	◌	◌	◌
HOS : Hunter Outcome Survey	◌	◌	◌

	Don't know it	Know it	Collaborate with it
iNTD Network – International Working Group on Neurotransmitter Related Disorders	○	○	○
International Collaborative Gaucher Group (ICGG) Gaucher Registry – DE	○	○	○
International Morquio A registry	○	○	○
International Niemann-Pick Disease Registry	○	○	○
LOVD: GAA gene (Glucosidase, alpha, acid) variant database	○	○	○
Mendelian cytogenetics network online database	○	○	○
MITOCON: National registry mitochondrial disease	○	○	○
Mucopolysaccharidosis type 1 (MPS I) Registry –	○	○	○
NCL-Registry: International neuronal ceroid lipofuscinoses patient registry	○	○	○
NCL-Registry: International neuronal ceroid lipofuscinoses patient registry	○	○	○
Neuro-Heart-Registry: Registry for patients with heart involment in neurovascular diseases	○	○	○
NMDcy: Cypriot Neuromuscular Diseases – contributes to TREAT-NMD	○	○	○
NMD-ES: Spanish Registry of Neuromuscular Diseases	○	○	○
Norvegian Porphyria Registry	○	○	○
Pompe Disease Registry – DE	○	○	○
Portuguese Fabry patient registry – contributing to the international Fabry registry	○	○	○
RAMEDIS : Rare Metabolic Diseases Database	○	○	○
RAMEDIS : Rare Metabolic Diseases mutation Database	○	○	○
Registry for Patients with Niemann-Pick Type C Disease	○	○	○
Registry for Wolfram syndrome, Alström syndrome, Bardet-Biedl syndrome	○	○	○

	Don't know it	Know it	Collaborate with it
and other rare diabetes syndromes – contributing to EURO-WABB			
Registry_Biobank Name	◌	◌	◌
The International Collaborative Gaucher Group (ICGG) Gaucher registry	◌	◌	◌
The international Fabry registry	◌	◌	◌
The international Mps I registry	◌	◌	◌
The international Pompe registry	◌	◌	◌
TREAT-NMD: Accelerating Treatments for Neuromuscular Diseases (registries)	◌	◌	◌
UK10K RARE NEUROMUSCULAR – Genetic neuromuscular diseases variant database	◌	◌	◌
X-ALD: X-linked adrenoleukodystrophy database	◌	◌	◌

*55. About scientific organisation, what is applicable for you?

	Don't know it	Know it	Collaborate with it
AECOM – Asociación Española para el Estudio de los Errores Congénitos del Metabolismo	◌	◌	◌
APS – Arbeitsgemeinschaft für Pädiatrische Stoffwechselstörungen (Working group for paediatric metabolic disturbances, Germany)	◌	◌	◌
B4B – Brains for Brain	◌	◌	◌
BIMDG – British Inherited Metabolic Disease Group	◌	◌	◌
CLIMB – Children Living with Inherited Metabolic Diseases, UK	◌	◌	◌

	Don't know it	Know it	Collaborate with it
EBC – European Brain Council	◌	◌	◌
ERNDIM Quality assurance in laboratory testing for IEM	◌	◌	◌
ESGCT – European Society of Gene and Cell Therapy	◌	◌	◌
EURORDIS – European Organisation for Rare Disorders	◌	◌	◌
Fondazione Telethon	◌	◌	◌
IBBS – International Brain Barriers Society	◌	◌	◌
IRDIRC	◌	◌	◌
ISNS – International Society for Neonatal Screening	◌	◌	◌
SEEIM – Sociedad Española de Errores Innatos del Metabolismo	◌	◌	◌
SFEIM – Societé Française pour l'Étude des Erreurs Innées du Métabolisme	◌	◌	◌
SIMD – Society for Inherited Metabolic Disorders	◌	◌	◌
SISMME – Societa' Italiana per lo Studio delle Malattie Metaboliche Ereditairie	◌	◌	◌
SPDM – Sociedade Portuguesa de Doenças Metabólicas	◌	◌	◌
SSIEM – The Society for Study of Inborn Errors of Metabolism	◌	◌	◌

*56. How many ongoing research do you participate in at your department at the moment.

[▾]

57. How many of these are clinical trials?

58. How many of the clinical trials are in phase I?

59. How many of the clinical trials are in phase II?

60. How many of the clinical trials are in phase III?

61. How many of the clinical trials are in phase IV?

European Reference Networks

Survey MetabERN for Universities

*1. Can you please type in your contact details

Name

Email Address

Phone Number

*2. Please select the name of your hospital /institute from the drop down menu

3. Please select what is applicable to your situation

- male
- female

4. Type of institution

- [ ] Public university
- [ ] Private university
- [ ] Public college
- [ ] Private college
- [ ] Research institute

*5. What is the legal status of your institution

- ○ Public
- ○ Private
- ○ mixed

*6. What is your position

- ○ Professor
- ○ Associate Professor
- ○ Lecturer
- ○ Senior Research Fellow
- ○ Postdoc/Research Fellow
- ○ Research Assistant
- ○ Teaching Assistant
- ○ Dean, head of department or other senior administration position
- ○ Other (please specify)

* 7. Please select the areas and fields in which your institution is undertaking research activities, please select all the ones of interest:

- Animal research
- Basic research
- Preclinical research
- Clinical research

Questions to inquire the level of activity in the research thematic area.

* 8. Please indicate which are, the main research activities performed inside your Institution

	No activity	Low activity	Rather low activity	Rather high activity	High activity
Health Systems Research					
Human and Social Science Research					
Epidemiology/Natural History of diseases					
Research in Genetics and Pathophysiology					
Pre-Clinical Therapeutic Research					
Therapeutic Research					
Research Platforms/Infrastructures					

	No activity	Low activity	Rather low activity	Rather high activity	High activity
Development and availability of tools needed to accelerate research on rare diseases	○	○	○	○	○

*9. **Academic** Health Systems Research

	No activity	Low activity	Rather low activity	Rather high activity	High activity
Research policy (e.g., guidelines development; national plans; health care resources)	○	○	○	○	○
Cost analysis (e.g., guidelines implementation)	○	○	○	○	○
New technologies (e.g., New diagnostic tests, population screening programmes, Innovative therapeutics)	○	○	○	○	○
Health-Technology assessment of Orphan Drugs	○	○	○	○	○
e-Health activities	○	○	○	○	○

*10. **Industrial** Health Systems Research

	No activity	Low activity	Rather low activity	Rather high activity	High activity
Research policy (e.g., guidelines development; national plans;	○	○	○	○	○

	No activity	Low activity	Rather low activity	Rather high activity	High activity
health care resources)					
Cost analysis (e.g., guidelines implementation)	◌	◌	◌	◌	◌
New technologies (e.g., New diagnostic tests, population screening programmes, Innovative therapeutics)	◌	◌	◌	◌	◌
Health-Technology assessment of Orphan Drugs	◌	◌	◌	◌	◌
e-Health activities	◌	◌	◌	◌	◌

* 11. Human and social science research

	No activity	Low activity	Rather low activity	Rather high activity	High activity
Society and RD (e.g., social perception, accessibility to care,…)	◌	◌	◌	◌	◌
RD, Research and Innovation (e.g., public/private cooperation, role of patients org,….)	◌	◌	◌	◌	◌
Care practices (self- management, health education policy,…)	◌	◌	◌	◌	◌
Patients awareness (e.g., understanding genetic data, importance of animal models,…)	◌	◌	◌	◌	◌
Support for caregivers	◌	◌	◌	◌	◌
Public Heath and RD (e.g., health care policies, prospective public policies,…)	◌	◌	◌	◌	◌

	No activity	Low activity	Rather low activity	Rather high activity	High activity
Methods for social support to improve quality of life	○	○	○	○	○
Services accessibility	○	○	○	○	○
Orphan Drugs availability	○	○	○	○	○
Methods to assess QoL	○	○	○	○	○

* 12. Epidemiology & Natural History of diseases

	No activity	Low activity	Rather low activity	Rather high activity	High activity
Registries	○	○	○	○	○
Databases/data management systems for epidemiology studies	○	○	○	○	○
Cohorts	○	○	○	○	○
Biomarkers to assess diagnosis, prognosis and severity of diseases	○	○	○	○	○
Diagnosis/prognosis methods (e.g., other than biomarkers)	○	○	○	○	○
Identification of new entities	○	○	○	○	○
Networks for ultra rare diseases	○	○	○	○	○

*13. Research in genetics and physiopathology

	No activity	Low activity	Rather low activity	Rather high activity	High activity
Gene identification and Molecular Mechanisms	○	○	○	○	○
New database method developments to be applied to omics research	○	○	○	○	○
Biobanking research	○	○	○	○	○
High throughput genomics/sequencing	○	○	○	○	○
New bioinformatic methods and developments	○	○	○	○	○
New technologies (e.g., atomic microscopy…)	○	○	○	○	○
Omics technology (e.g., proteo, metabolo..)	○	○	○	○	○
Development of new animal models	○	○	○	○	○
New Imaging/Microscopy methods	○	○	○	○	○
Development of new therapeutic tools	○	○	○	○	○

*14. Pre-clinical therapeutic research

	No activity	Low activity	Rather low activity	Rather high activity	High activity
Proof-of-concept	○	○	○	○	○
Drug libraries	○	○	○	○	○

	No activity	Low activity	Rather low activity	Rather high activity	High activity
Search for active chemical compounds					
High throughput drug screening					
Therapeutic target identification/validation (e.g.,. stem cells, systems biology)					
Cell therapy research (e.g., viral vectors…)					
Innovative biotechnological research (e.g., monoclonal antibodies, cell and gene therapy, enzyme therapy…)					
Drug toxicology/pharmacology					

*15. Therapeutic research

	No activity	Low activity	Rather low activity	Rather high activity	High activity
Drug development					
Phase I/II Clinical Trials					
Bioproduction centres					
Epidemiological/clinical/ therapeutical investigations					
Medical devices					
Assesment of surgery procedures					
Transplants					

	No activity	Low activity	Rather low activity	Rather high activity	High activity
Pharma/academic partnerships	○	○	○	○	○

*16. Platforms / Infrastructures development

	No activity	Low activity	Rather low activity	Rather high activity	High activity
Bioinformatics	○	○	○	○	○
Pharmacogenomic diagnostic assays	○	○	○	○	○
Population Registries	○	○	○	○	○
Genetically-modified animal model repositories	○	○	○	○	○
GMP platforms	○	○	○	○	○
Biobanking	○	○	○	○	○
Genomic and proteomic platforms	○	○	○	○	○
Long-term cohort of patients studies	○	○	○	○	○
High throughput drug screening	○	○	○	○	○

*17. Human resources

	No activity	Low activity	Rather low activity	Rather high activity	High activity
Rotational positions for clinicians	◌	◌	◌	◌	◌
Rotational positions for researchers	◌	◌	◌	◌	◌
Specific training in RD	◌	◌	◌	◌	◌
Training in bioinformatics	◌	◌	◌	◌	◌

*18. Development and availability of tools needed to accelerate research on rare disease.

	No activity	Low activity	Rather low activity	Rather high activity	High activity
Network development	◌	◌	◌	◌	◌
Creation of transnational research consortiums	◌	◌	◌	◌	◌
Transnational calls for projects funding	◌	◌	◌	◌	◌
Opening of national research programmes to international collaboration	◌	◌	◌	◌	◌
Transnational research infrastructures	◌	◌	◌	◌	◌
Development of an inventory/directory of databases of experts	◌	◌	◌	◌	◌
Transnational collection of funded RD research projects	◌	◌	◌	◌	◌

*19. What influences and governs research at your institution?

	Strongly agree	Agree to some extent	Disagree to some extent	Strongly disagree	No information available	Not applicable
Ministry/ national research agency has a strong say	○	○	○	○	○	○
Central university level mostly decides	○	○	○	○	○	○
Faculties/ departments/ institutes decide very much on what they do	○	○	○	○	○	○
Individual professors/ Researchers do very much what they want	○	○	○	○	○	○
In my own position, I can influence research agendas at my faculty/department/ institute	○	○	○	○	○	○

*20. Please rate the importance of funding sources for research

	Very important	Of some importance	Not important	Do not know	Not applicable
Institutional funding by government	○	○	○	○	○
Research grants (EU)	○	○	○	○	○
Other international research grants	○	○	○	○	○
Contracts from industry	○	○	○	○	○
Endowment income, donations	○	○	○	○	○
Research Board	○	○	○	○	○

	Very important	Of some importance	Not important	Do not know	Not applicable
Charity	○	○	○	○	○
Private company	○	○	○	○	○
Scientific Society/PatientsOrganization	○	○	○	○	○
Local Istitutional Funding by Single Univeristies,	○	○	○	○	○
Local and/or Regional authorities,	○	○	○	○	○
Funding by Health Authorities	○	○	○	○	○
Funding by no-profit, Private Agencies (e.g. Telethon),	○	○	○	○	○

*21. Who decides on how this budget is allocated to the faculties/ departments?

- ○ Institutional leadership (Dean, president, director)
- ○ A central body of the institution (academic senate or similar)
- ○ Ministry/ national agency

*22. How would you generally describe facilities and infrastructure at your institution

	International standard or close to it	Good national standard	Insufficient	Do not know	Not applicable

	International standard or close to it	Good national standard	Insufficient	Do not know	Not applicable
Research support services / unit					
Research laboratories					
Teaching labs (if different from research Labs)					
Large research equipment that requires technicians					
Scientific databases (e.g. ISI Web of Knowledge)					
Knowledge Transfer Office					
Science Park					
Research ethics committee					
Libraries					
Research publication repositories					
Computer equipment	C				
IT Services					
Accommodation for visiting scholars					
Buildings/premi ses in general					
Power supply/ electricity					
Communication infrastructure (phone, internet etc.)					

*23. Does your institution have, or engages in, any of the following

	Yes very strong	Yes occasionally / some	No	Do not know	Not applicable
Spin-offs	○	○	○	○	○
Research collaboration with industry	○	○	○	○	○
International research collaboration	○	○	○	○	○
Research collaboration with public/ state offices and agencies	○	○	○	○	○
Patents	○	○	○	○	○

*24. Has your institution been involved in applications for research projects (regardless whether successful or not)?

	Frequently as an applicant	Frequently as a partner	Occasionally as an applicant	Occasionally as partner	No involvement of application
National, involving colleagues from another institution	○	○	○	○	○
Horizon 2020 / FP7	○	○	○	○	○
Other European project	○	○	○	○	○
Other International project	○	○	○	○	○

* 25. When have you been last research active as a health professional?

(Research activity is defined here as being involved in the design or implementation of a research project either as a researcher or a supervisor)

- ○ Within the last 2 years
- ○ More than 2 years ago
- ○ Never been research active as a health professional

* 26. Have you published any of the following over the last 3 years 2014-2017?

	Yes mostly with single authorship	Yes mostly co-authored	Only supervising	No
ISI journal articles	○	○	○	○
Other journal articles (non-ISI)	○	○	○	○
Papers presented at international conferences	○	○	○	○
Papers presented at national conferences	○	○	○	○
PhD. Thesis	○	○	○	○
Books	○	○	○	○
Book chapters	○	○	○	○
Reports	○	○	○	○

*27. If co-authored, who do you write with more often?

	Colleagues from my institution	Colleagues from other institutions	European Colleagues	Intercontinental Colleagues	No co-authoring
ISI journal articles	◯	◯	◯	◯	◯
Other journal articles (non-ISI)	◯	◯	◯	◯	◯
Papers presented at international conferences	◯	◯	◯	◯	◯
Papers presented at national conferences	◯	◯	◯	◯	◯
PhD. Thesis	◯	◯	◯	◯	◯
Books	◯	◯	◯	◯	◯
Book chapters	◯	◯	◯	◯	◯
Reports	◯	◯	◯	◯	◯

*28. What factors had motivated you to be research active?

	Did not motivate me	Yes some motivation	very strong motivation	N/A
Personal motivation', '	◯	◯	◯	◯
Completing an academic degree'	◯	◯	◯	◯

	Did not motivate me	Yes some motivation	very strong motivation	N/A
Professional development	○	○	○	○
Career advancement	○	○	○	○
Part of the professional role	○	○	○	○
For publication	○	○	○	○
To gain more expertise	○	○	○	○

*29. If not active in research define the potential barriers to research engagement:

If active in research: Is there anything you can point out that prevents or discourages you from engaging in research.

- ☐ Not enough time available to engage in research
- ☐ Clinical workload pressures
- ☐ Lack of support/encouragement
- ☐ Lack of funding
- ☐ Lack of available resources for research
- ☐ Difficulties gaining ethical approval
- ☐ Research not valued in organisation
- ☐ Weaknesses in research skills
- ☐ Personal circumstances (e.g., family commitments limiting time for research)
- ☐ Lack of research opportunities
- ☐ Lack of supervision/mentorship

- [ ] Difficulties collecting or gaining access to data
- [ ] There is no reason why people should be limited or not engaged in research

*30. What are the main problems that researchers face at your institution

	Strongly agree	Agree to some extent	Disagree to some extend	Strongly disagree	Do not know	N/A
There is no or insufficient research funding	○	○	○	○	○	○
There are no or insufficient research facilities	○	○	○	○	○	○
Some research facilities can only be accessed by some researchers	○	○	○	○	○	○
There is no or insufficient administrative capacity to prepare research proposals, manage research projects etc.)	○	○	○	○	○	○
Most researchers have no international research contacts	○	○	○	○	○	○
Most researchers are not internationally mobile	○	○	○	○	○	○
It is not possible to hire international researchers	○	○	○	○	○	○
Most researchers have to teach, and cannot put enough focus on their research	○	○	○	○	○	○
Most researchers have other jobs on the side and cannot put enough focus on their research	○	○	○	○	○	○

*31. What do you think are the reasons why people in your Institution are not, or limited engaged in research?

- [ ] Not enough time available to engage in research
- [ ] Clinical workload pressures
- [ ] Lack of support/encouragement
- [ ] Lack of funding
- [ ] Lack of available resources for research
- [ ] Difficulties gaining ethical approval
- [ ] Research not valued in organisation
- [ ] Weaknesses in research skills
- [ ] Personal circumstances (e.g., family commitments limiting time for research)
- [ ] Lack of research opportunities
- [ ] Lack of supervision/mentorship
- [ ] Difficulties collecting or gaining access to data
- [ ] There is no reason why people should be limited or not engaged in research

*32. What do you see as the main problems for research in your **country**?

	strongly agree	agree to some extent	neither agree nor disagree	disagree	strongly disagree	Do not know
Research is not given sufficiently high priority by the state	○	○	○	○	○	○
There is no national strategy, which would provide a clear framework	○	○	○	○	○	○
There is no or insufficient research funding	○	○	○	○	○	○

	strongly agree	agree to some extent	neither agree nor disagree	disagree	strongly disagree	Do not know
There are no or insufficient research facilities	○		○	○	○	○
Research is too much concentrated in a few institutions	○	○	○	○	○	○
Higher education institutions are focusing too much on teaching, and neglect research	○	○	○	○	○	○
Research is not sufficiently applied, and ignores the needs of the industry and society	○	○	○	○	○	○
There is too much focus on applied research	○	○	○	○	○	○
Interdisciplinary research is not sufficiently encouraged	○	○	○	○	○	○
The best researchers are leaving the country, which is a problem	○	○	○	○		○
The best researchers are leaving the country, but they still continue to Contribute to national research	○	○	○	○	○	○
Young researchers are leaving the country	○	○	○	○	○	○
Young talented people look for careers outside of the research and higher Education sectors	○	○	○	○	○	○

*33. Of the changes listed in the table below, indicate how effective you believe they would for increasing the level of research activity in your Institution

	Would have no effect	Would be somewhat effective	Would be greatly effective

	Would have no effect	Would be somewhat effective	Would be greatly effective
Increased protected working time for research	◯	◯	◯
Higher levels of ongoing research training	◯	◯	◯
Recruiters in the health service giving more value to research experience when selecting candidates for clinical positions	◯	◯	◯
A more simplified and efficient ethics application process	◯	◯	◯
Greater levels of mentorship and support when conducting research	◯	◯	◯
More opportunities to advance one’s research qualifications (e.g. post-doctoral fellowships)	◯	◯	◯
More funded research posts	◯	◯	◯
More opportunities for collaboration with other clinical or academic researchers	◯	◯	◯

*34. What is applicable for you in the following list about research infrastructure

	Don't know it	Know it	Collaborate with it
BBMRI Biobanking and Biomolecular Resources Research Infrastructure	◯	◯	◯
C-ERIC Central-European Research Infrastructures Consortium	◯	◯	◯
CLARIN Common Language Resources and Technology Infrastructure	◯	◯	◯
EATRIS European Advanced Translational Research Infrastructure in Medicine	◯	◯	◯

	Don't know it	Know it	Collaborate with it
ECRIN European Clinical Research Infrastructure Network	◌	◌	◌
ELIXIR The European Life-Science Infrastructure for Biological Information	◌	◌	◌
ERA European Research Area	◌	◌	◌
ERIC European Research Infrastructure Consortium	◌	◌	◌
ESFRI European Strategy Forum on Research Infrastructure	◌	◌	◌
EU-OPENSCREEN European Infrastructure of Open Screening Platforms for Chemical Biology	◌	◌	◌
Euro-BioImaging European Research Infrastructure for Imaging Technologies in Biological and Biomedical Sciences	◌	◌	◌
INFRAFRONTIER European Research Infrastructure for the generation, phenotyping, archiving and distribution of mouse	◌	◌	◌
ISBE Infrastructure for Systems Biology Europe	◌	◌	◌
MIRRI Microbial Resource Research Infrastructure disease models	◌	◌	◌
NRRI Research Infrastructure Roadmap	◌	◌	◌
PRACE Partnership for Advanced Computing in Europe	◌	◌	◌
RI Research infrastructure(s)	◌	◌	◌

*35. What is applicable for you in the following list about biobanks

	Don't know it	Know it	Collaborate with it
Basque Biobank	◌	◌	◌

	Don't know it	Know it	Collaborate with it
Biobanco.pt (Portuguese National Consortium of Biobanks)	○	○	○
Biobank Inherited Metabolic Disorders (blood/urine/liquor/fibroblasts)	○	○	○
Biobank of the Estonian genome center	○	○	○
BioNER: Biobank of the Institute of Rare Diseases Research/Institute of Health Carlos III	○	○	○
Brain-net – brain tissue bank Aachen	○	○	○
Brain-Net – brain tissue bank Munster (pediatric neuropathology)	○	○	○
Brain-Net Germany : National network of brain banks in Germany	○	○	○
Cell line and DNA Biobank from patients affected by genetic diseases	○	○	○
CIBERER Biobank (Biobank of rare diseases)	○	○	○
DNA biobank of the Department of Molecular Genetics and Diagnostics, National Institute of Environmental Health (NIEH) (EuroBioBank partner)	○	○	○
Genetic alliance Registry and Biobank	○	○	○
EuroBioBank European Network of DNA, cell and tissue banks for Rare Diseases	○	○	○
Human DNA and cell biobank of Genethon – genetic diseases, mainly neuromuscular diseases – part of the EuroBioBank network	○	○	○
Human tissue biobank of Neuromuscular diseases (EuroBioBank partner)	○	○	○
Neuromuscular Tissue Bank (NMTB) (EuroBioBank partner)	○	○	○
Telethon Network of Genetic Biobanks	○	○	○
UK Biobank	○	○	○

*36. What is applicable for you in the following list about registry and database

	Don't know it	Know it	Collaborate with it
(NCARDRS) National Congenital Anomaly and Rare Disease Registration Service	○	○	○
Austrian Country Node of the Human Variome Project (HVP)	○	○	○
Austrian registry for inborn errors of metabolism	○	○	○
Belgian Neuromuscular Disease Registry	○	○	○
Belgian patient database for Wilson disease – contributes to the EuroWilson registry (terminated)	○	○	○
Biobank Inherited Metabolic Disorders (blood/urine/liquor/fibroblasts)	○	○	○
Canadian Neuromuscular Disease Registry – part of the TREAT-NMD network	○	○	○
DDRMD – Dutch Diagnosis Registration Metabolic Diseases	○	○	○
Dutch Neuromuscular Database CRAMP: Computer Registry of All Myopathies and Polyneuropathies	○	○	○
Dutch patient registry for Fabry disease	○	○	○
Dutch patient registry for Gaucher disease	○	○	○
Dutch patient registry for Niemann-Pick Disease Type B	○	○	○
Dutch patient registry for Niemann-Pick Disease Type C	○	○	○
ECARUCA: cytogenetic and clinical database on rare chromosomal disorders	○	○	○
E-HOD European Network and Registry for Homocystinurias and Methylation Defects	○	○	○
EIMD: European registry and network for intoxication type metabolic diseases	○	○	○

	Don't know it	Know it	Collaborate with it
English alpha-1 antitrypsin deficiency registry – contributes to the Alpha One International Registry (AIR)	◯	◯	◯
English cystinosis registry	◯	◯	◯
English hyperoxaluria registry	◯	◯	◯
English phenylketonuria registry	◯	◯	◯
EPNET: European porphyria Network	◯	◯	◯
E-RARE Consortium	◯	◯	◯
ESNEK – Rare paediatric neurological disease registry Germany	◯	◯	◯
EUNEFRON: registry of the European network for the study of orphan nephropathies	◯	◯	◯
EUROCAT European Surveillance on congenital anomalies	◯	◯	◯
EUROGLYCANET – International patient registry and cohort for congenital disorders of glycosylation	◯	◯	◯
European cystinosis cohort	◯	◯	◯
European Porphyria Registry (EPR)	◯	◯	◯
Fabry Disease Registry –DE	◯	◯	◯
FOS : Fabry Outcome Survey	◯	◯	◯
French certified registry of glycogen storage disease type 2	◯	◯	◯
French certified registry of patients affected by Gaucher disease	◯	◯	◯
French cystinosis registry	◯	◯	◯
French registry of neuromuscular diseases from reference centres	◯	◯	◯
Galactosemia Patient Registry – part of the Galactosemia Network	◯	◯	◯

	Don't know it	Know it	Collaborate with it
Genetic alliance Registry and Biobank	○	○	○
German mucopolysaccharidosis patient registry	○	○	○
HOS : Hunter Outcome Survey	○	○	○
iNTD Network – International Working Group on Neurotransmitter Related Disorders	○	○	○
International Collaborative Gaucher Group (ICGG) Gaucher Registry – DE	○	○	○
International Morquio A registry	○	○	○
International Niemann-Pick Disease Registry	○	○	○
LOVD: GAA gene (Glucosidase, alpha, acid) variant database	○	○	○
Mendelian cytogenetics network online database	○	○	○
MITOCON: National registry mitochondrial disease	○	○	○
Mucopolysaccharidosis type 1 (MPS I) Registry –	○	○	○
NCL-Registry: International neuronal ceroid lipofuscinoses patient registry	○	○	○
NCL-Registry: International neuronal ceroid lipofuscinoses patient registry	○	○	○
Neuro-Heart-Registry: Registry for patients with heart involment in neurovascular diseases	○	○	○
NMDcy: Cypriot Neuromuscular Diseases – contributes to TREAT-NMD	○	○	○
NMD-ES: Spanish Registry of Neuromuscular Diseases	○	○	○
Norvegian Porphyria Registry	○	○	○
Pompe Disease Registry – DE	○	○	○
Portuguese Fabry patient registry – contributing to the international Fabry registry	○	○	○

	Don't know it	Know it	Collaborate with it
RAMEDIS : Rare Metabolic Diseases Database	○	○	○
RAMEDIS : Rare Metabolic Diseases mutation Database	○	○	○
Registry for Patients with Niemann-Pick Type C Disease	○	○	○
Registry for Wolfram syndrome, Alström syndrome, Bardet-Biedl syndrome and other rare diabetes syndromes – contributing to EURO-WABB	○	○	○
Registry_Biobank Name	○	○	○
The International Collaborative Gaucher Group (ICGG) Gaucher registry	○	○	○
The international Fabry registry	○	○	○
The international Mps I registry	○	○	○
The international Pompe registry	○	○	○
TREAT-NMD: Accelerating Treatments for Neuromuscular Diseases (registries)	○	○	○
UK10K RARE NEUROMUSCULAR – Genetic neuromuscular diseases variant database	○	○	○
X-ALD: X-linked adrenoleukodystrophy database	○	○	○

*37. What is applicable for you in the following list about scientific organisation

	Don't know it	Know it	Collaborate with it
AECOM – Asociación Española para el Estudio de los Errores Congénitos del Metabolismo	○	○	○

	Don't know it	Know it	Collaborate with it
APS – Arbeitsgemeinschaft für Pädiatrische Stoffwechselstörungen (Working group for paediatric metabolic disturbances, Germany)			
B4B – Brains for Brain			
BIMDG – British Inherited Metabolic Disease Group			
CLIMB – Children Living with Inherited Metabolic Diseases, UK			
EBC – European Brain Council			
ERNDIM Quality assurance in laboratory testing for IEM			
ESGCT – European Society of Gene and Cell Therapy			
EURORDIS – European Organisation for Rare Disorders			
Fondazione Telethon			
IBBS – International Brain Barriers Society			
IRDIRC			
ISNS – International Society for Neonatal Screening			
SEEIM – Sociedad Española de Errores Innatos del Metabolismo			
SFEIM – Societé Française pour l'Étude des Erreurs Innées du Métabolisme			
SIMD – Society for Inherited Metabolic Disorders			
SISMME – Societa' Italiana per lo Studio delle Malattie Metaboliche Ereditairie			
SPDM – Sociedade Portuguesa de Doenças Metabólicas			
SSIEM – The Society for Study of Inborn Errors of Metabolism			

-

Survey MetabERN for Laboratories

PAGE TITLE

* 1. Can you please type in your contact details

Name

Email Address

Phone Number

* 2. Please select the name of your hospital /institute from the drop down menu

3. Please select what is applicable to your situation

- ○ male
- ○ female

4. Academic degrees (if you have several, just the once that are most important for your researcher career)

	Year of award	Country of award
Undergraduate degree (or equivalent)	Undergraduate degree (or equivalent) Year of award menu	Undergraduate degree (or equivalent) Country of award menu
Master degree (or equivalent)	Master degree (or equivalent) Year of award menu	Master degree (or equivalent) Country of award menu
Doctorate	Doctorate Year of award menu	Doctorate Country of award menu
Specialization Degree	Specialization Degree Year of award menu	Specialization Degree Country of award menu

5. Type of institution

- [ ] Public university
- [ ] Private university
- [ ] Public college
- [ ] Private college
- [ ] Research institute

*6. What is your position

- ( ) Professor
- ( ) Associate Professor
- ( ) Lecturer
- ( ) Senior Research Fellow
- ( ) Postdoc/Research Fellow
- ( ) Research Assistant
- ( ) Teaching Assistant
- ( ) Dean, head of department or other senior administration position
- ( ) Other (please specify)

7. What type of research do you perform?

- [ ] Animal research
- [ ] Basic research
- [ ] Preclinical research

☐ Clinical research

*8. Please indicate which are, the main research activities performed inside your Institution

	No activity	Low activity	Rather low activity	Rather high activity	High activity
Health Systems Research	○	○	○	○	○
Human and Social Science Research	○	○	○	○	○
Epidemiology/Natural History of diseases	○	○	○	○	○
Research in Genetics and Pathophysiology	○	○	○	○	○
Pre-Clinical Therapeutic Research	○	○	○	○	○
Therapeutic Research	○	○	○	○	○
Research Platforms/Infrastructures	○	○	○	○	○
Development and availability of tools needed to accelerate research on rare diseases	○	○	○	○	○

*9. **Academic** Health Systems Research

	No activity	Low activity	Rather low activity	Rather high activity	High activity
Research policy (e.g., guidelines development; national plans; health care resources)	○	○	○	○	○
Cost analysis (e.g., guidelines implementation)	○	○	○	○	○

	No activity	Low activity	Rather low activity	Rather high activity	High activity
New technologies (e.g., New diagnostic tests, population screening programmes, Innovative therapeutics)	◌	◌	◌	◌	◌
Health-Technology assessment of Orphan Drugs	◌	◌	◌	◌	◌
e-Health activities	◌	◌	◌	◌	◌

*10. **Industrial** Health Systems Research

	No activity	Low activity	Rather low activity	Rather high activity	High activity
Research policy (e.g., guidelines development; national plans; health care resources)	◌	◌	◌	◌	◌
Cost analysis (e.g., guidelines implementation)	◌	◌	◌	◌	◌
New technologies (e.g., New diagnostic tests, population screening programmes, Innovative therapeutics)	◌	◌	◌	◌	◌
Health-Technology assessment of Orphan Drugs	◌	◌	◌	◌	◌
e-Health activities	◌	◌	◌	◌	◌

*11. Human and social science research

	No activity	Low activity	Rather low activity	Rather high activity	High activity
Society and RD (e.g., social perception, accessibility to care,…)	◌	◌	◌	◌	◌
RD, Research and Innovation (e.g., public/private cooperation, role of patients org,….)	◌	◌	◌	◌	◌
Care practices (self- management, health education policy,…)	◌	◌	◌	◌	◌
Patients awareness (e.g., understanding genetic data, importance of animal models,…)	◌	◌	◌	◌	◌
Support for caregivers	◌	◌	◌	◌	◌
Public Heath and RD (e.g., health care policies, prospective public policies,…)	◌	◌	◌	◌	◌
Methods for social support to improve quality of life	◌	◌	◌	◌	◌
Services accessibility	◌	◌	◌	◌	◌
Orphan Drugs availability	◌	◌	◌	◌	◌
Methods to assess QoL	◌	◌	◌	◌	◌

* 12. Epidemiology & Natural History of diseases

	No activity	Low activity	Rather low activity	Rather high activity	High activity
Registries	◌	◌	◌	◌	◌
Databases/data management systems for	◌	◌	◌	◌	◌

	No activity	Low activity	Rather low activity	Rather high activity	High activity
epidemiology studies					
Cohorts	◌	◌	◌	◌	◌
Biomarkers to assess diagnosis, prognosis and severity of diseases	◌	◌	◌	◌	◌
Diagnosis/prognosis methods (e.g., other than biomarkers)	◌	◌	◌	◌	◌
Identification of new entities	◌	◌	◌	◌	◌
Networks for ultra rare diseases	◌	◌	◌	◌	◌

*13. Research in genetics and physiopathology

	No activity	Low activity	Rather low activity	Rather high activity	High activity
Gene identification and Molecular Mechanisms	◌	◌	◌	◌	◌
New database method developments to be applied to omics research	◌	◌	◌	◌	◌
Biobanking research	◌	◌	◌	◌	◌
High throughput genomics/sequencing	◌	◌	◌	◌	◌
New bioinformatic methods and developments	◌	◌	◌	◌	◌
New technologies (e.g., atomic microscopy…)	◌	◌	◌	◌	◌
Omics technology (e.g., proteo, metabolo..)	◌	◌	◌	◌	◌

	No activity	Low activity	Rather low activity	Rather high activity	High activity
Development of new animal models					
New Imaging/Microscopy methods					
Development of new therapeutic tools					

* 14. Pre-clinical therapeutic research

	No activity	Low activity	Rather low activity	Rather high activity	High activity
Proof-of-concept					
Drug libraries					
Search for active chemical compounds					
High throughput drug screening					
Therapeutic target identification/validation (e.g.,. stem cells, systems biology)					
Cell therapy research (e.g., viral vectors…)					
Innovative biotechnological research (e.g., monoclonal antibodies, cell and gene therapy, enzyme therapy…)					
Drug toxicology/pharmacology					

*15. Therapeutic research

	No activity	Low activity	Rather low activity	Rather high activity	High activity
Drug development	○	○	○	○	○
Phase I/II Clinical Trials	○	○	○	○	○
Bioproduction centres	○	○	○	○	○
Epidemiological/clinical/ therapeutical investigations	○	○	○	○	○
Medical devices	○	○	○	○	○
Assesment of surgery procedures	○	○	○	○	○
Transplants	○	○	○	○	○
Pharma/academic partnerships	○	○	○	○	○

*16. Platforms / Infrastructures development

	No activity	Low activity	Rather low activity	Rather high activity	High activity
Bioinformatics	○	○	○	○	○
Pharmacogenomic diagnostic assays	○	○	○	○	○
Population Registries	○	○	○	○	○
Genetically-modified animal model	○	○	○	○	○

	No activity	Low activity	Rather low activity	Rather high activity	High activity
repositories					
GMP platforms	◌	◌	◌	◌	◌
Biobanking	◌	◌	◌	◌	◌
Genomic and proteomic platforms	◌	◌	◌	◌	◌
Long-term cohort of patients studies	◌	◌	◌	◌	◌
High throughput drug screening	◌	◌	◌	◌	◌

* 17. Human resources

	No activity	Low activity	Rather low activity	Rather high activity	High activity
Rotational positions for clinicians	◌	◌	◌	◌	◌
Rotational positions for researchers	◌	◌	◌	◌	◌
Specific training in RD	◌	◌	◌	◌	◌
Training in bioinformatics	◌	◌	◌	◌	◌

* 18. Development and availability of tools needed to accelerate research on rare disease.

	No activity	Low activity	Rather low activity	Rather high activity	High activity
Network development	◌	◌	◌	◌	◌
Creation of transnational research consortiums	◌	◌	◌	◌	◌
Transnational calls for projects funding	◌	◌	◌	◌	◌
Opening of national research programmes to international collaboration	◌	◌	◌	◌	◌
Transnational research infrastructures	◌	◌	◌	◌	◌
Development of an inventory/directory of databases of experts	◌	◌	◌	◌	◌
Transnational collection of funded RD research projects	◌	◌	◌	◌	◌

* 19. How do you usually conduct your research activities

	Never	Seldom	Sometimes	Very commonly	N/A
Alone	◌	◌	◌	◌	◌
Within a research group/team	◌	◌	◌	◌	◌
Collaboration with colleagues from my institution	◌	◌	◌	◌	◌
Collaboration with colleagues from other institutions in my country	◌	◌	◌	◌	◌
Collaboration with international colleagues at EU level	◌	◌	◌	◌	◌
Collaboration with intercontinental colleagues	◌	◌	◌	◌	◌
Collaboration with domestic industry/Biotech Company	◌	◌	◌	◌	◌

	Never	Seldom	Sometimes	Very commonly	N/A
Collaboration with international industry/Biotech Company					
Collaboration with local Patients Organizations					
Collaboration with international Patients Organizations					
Collaboration with local Scientific Organizations					
Collaboration with international					
Scientific Organizations					

*20. Do you have access to the following? If yes, how would you rate the quality?

	International standard or close to it	Good national standard	Insufficient	Do not know	Not applicable
Research support services / unit					
Research laboratories					
Teaching labs (if different from research Labs)					
Large research equipment that requires technicians					
Scientific databases (e.g. ISI Web of Knowledge)					
Knowledge Transfer Office					

	International standard or close to it	Good national standard	Insufficient	Do not know	Not applicable
Science Park	◌	◌	◌	◌	◌
Research ethics committee	◌	◌	◌	◌	◌
Libraries	◌	◌	◌	◌	◌
Research publication repositories	◌	◌	◌	◌	◌
Computer equipment	◌	◌	◌	◌	◌
IT Services	◌	◌	◌	◌	◌
Accommodation for visiting scholars	◌	◌	◌	◌	◌
Buildings/premi ses in general	◌	◌	◌	◌	◌
Power supply/ electricity	◌	◌	◌	◌	◌
Communication infrastructure (phone, internet etc.)	◌	◌	◌	◌	◌

*21. Which of the following describes your situation

	Strongly agree	Agree to some extent	Disagree to some extend	Strongly disagree	Do not know	N/A
I have access to the necessary research infrastructure (labs,	◌	◌	◌	◌	◌	◌
computers, library etc.) to do my research	◌	◌	◌	◌	◌	◌
I have the necessary staff support to do my research	◌	◌	◌	◌	◌	◌

	Strongly agree	Agree to some extent	Disagree to some extend	Strongly disagree	Do not know	N/A
I have the necessary funding to do my research	○	○	○	○	○	○
In my position, I have a high level of autonomy in developing my research	○	○	○	○	○	○
I am involved in interdisciplinary research collaboration within my institution	○	○	○	○	○	○
I am involved in interdisciplinary research collaboration within colleagues from other institutions	○	○	○	○	○	○
I am involved in spin-off activities, research incubators or similar	○	○	○	○	○	○
I am/have been involved in patents	○	○	○	○	○	○
I frequently attend international conferences	○	○	○	○	○	○
I am very dedicated to my work	○	○	○	○	○	○
I am satisfied with my career	○	○	○	○	○	○
I am satisfied with my salary	○	○	○	○	○	○
I am proud to work at my institution/organisation	○	○	○	○	○	○
My working situation is difficult, due to a high workload	○	○	○	○	○	○
My working situation is difficult, due to bureaucracy, administration	○	○	○	○	○	○

*22. Have you published any of the following over the last 3 years 2014-2017?

	Yes mostly with single authorship	Yes mostly co-authored	Only supervising	No
ISI journal articles	◌	◌	◌	◌
Other journal articles (non-ISI)	◌	◌	◌	◌
Papers presented at international conferences	◌	◌	◌	◌
Papers presented at national conferences	◌	◌	◌	◌
PhD. Thesis	◌	◌	◌	◌
Books	◌	◌	◌	◌
Book chapters	◌	◌	◌	◌
Reports	◌	◌	◌	◌

*23. If co-authored, who do you write with more often?

	Colleagues from my institution	Colleagues from other institutions	European Colleagues	Intercontinental Colleagues	No co-authoring
ISI journal articles	◌	◌	◌	◌	◌
Other journal articles (non-ISI)	◌	◌	◌	◌	◌
Papers presented at international conferences	◌	◌	◌	◌	◌
Papers presented at national conferences	◌	◌	◌	◌	◌
PhD. Thesis	◌	◌	◌	◌	◌

	Colleagues from my institution	Colleagues from other institutions	European Colleagues	Intercontinental Colleagues	No co-authoring
Books	○	○	○	○	○
Book chapters	○	○	○	○	○
Reports	○	○	○	○	○

*24. Have you been involved in applications for research projects (regardless wether successful or not)?

	Frequently as an applicant	Frequently as a partner	Occasionally as an applicant	Occasionally as partner	No involvement of application
National, involving colleagues from another institution	○	○	○	○	○
Horizon 2020 / FP7	○	○	○	○	○
Other European project	○	○	○	○	○
Other International project	○	○	○	○	○

*25. Have you ever worked as a researcher for the following period?

	Less than 1 year	Between 1 and 2 years	More than 2 years
In your country	○	○	○

	Less than 1 year	Between 1 and 2 years	More than 2 years
In the EU (outside your country)	◌	◌	◌
In the US	◌	◌	◌
both in the EU and the US	◌	◌	◌
neither in the EU nor in the US	◌	◌	◌

*26. Evaluate your research skills using the table below

	very weak	weak	average strong	strong	very strong	don't know
Handling routine data	◌	◌	◌	◌	◌	◌
Generating a research idea	◌	◌	◌	◌	◌	◌
Conducting a literature review	◌	◌	◌	◌	◌	◌
Applying for ethics approval	◌	◌	◌	◌	◌	◌
Applying for funding	◌	◌	◌	◌	◌	◌
Designing quantitative research	◌	◌	◌	◌	◌	◌
Designing qualitative research	◌	◌	◌	◌	◌	◌
Recruiting participants	◌	◌	◌	◌	◌	◌
Collecting data	◌	◌	◌	◌	◌	◌
Quantitative data analysis	◌	◌	◌	◌	◌	◌
Qualitative data analysis	◌	◌	◌	◌	◌	◌

	very weak	weak	average strong	strong	very strong	don't know
Presenting the results	○	○	○	○	○	○
Critical appraisal of research	○	○	○	○	○	○
Orally presenting research	○	○	○	○	○	○
Publishing research	○	○	○	○	○	○
Teaching colleagues and/or students	○	○	○	○	○	○
Working as a member of a team doing research	○	○	○	○	○	○
Accessing resources to undertake research (e.g, money, information, equipment)	○	○	○	○	○	○
Undertaking administrative activities	○	○	○	○	○	○

*27. How do you rate the following in relation to the motivation to do research abroad?

	not important	quite important	important	very important	extremely important
Learn different Language	○	○	○	○	○
Career progression goals	○	○	○	○	○
Access to knowledge not	○	○	○	○	○
available in your country	○	○	○	○	○
Getting access to the facilities / equipment necessary	○	○	○	○	○
to your research	○	○	○	○	○
Opportunities to collaborate	○	○	○	○	○

	not important	quite important	important	very important	extremely important
with top-class researchers	○	○	○	○	○
More attractive remuneration	○	○	○	○	○
schemes	○	○	○	○	○
More opportunities to work in the	○	○	○	○	○
industry	○	○	○	○	○
Obtaining funding for own research	○	○	○	○	○
Availability of adequate schools you're your children	○	○	○	○	○
Work opportunities for your partner (and other family members)	○	○	○	○	○
Quality and cost of accommodation	○	○	○		○
Personal interest in the culture of foreign country	○	○	○	○	○

*28. Please rank the effect of the international mobility on your research career

	remained unchanged	a little bit improved	improved	strongly improved	N/A
Publication output	○	○	○	○	○
Patient output	○	○	○	○	○
Ability to work in the industrial sector	○	○	○	○	○

	remained unchanged	a little bit improved	improved	strongly improved	N/A
Access to infrastructure and know-how	◌	◌	◌	◌	◌
Access to an international network of professionals active in your field	◌	◌	◌	◌	◌
Professional experience as researcher	◌	◌	◌	◌	◌
Future job opportunities in the country where you have previously worked/studied	◌	◌	◌	◌	◌
"Recognition" in the research community as a researcher	◌	◌	◌	◌	◌

*29. What are the main problems that researchers face at your **institution**?

	Strongly agree	Agree to some extent	Disagree to some extend	Strongly disagree	Do not know	N/A
There is no or insufficient research funding	◌	◌	◌	◌	◌	◌
There are no or insufficient research facilities	◌	◌	◌	◌	◌	◌
Some research facilities can only be accessed by some researchers	◌	◌	◌	◌	◌	◌
There is no or insufficient administrative capacity to prepare research proposals, manage research projects etc.)	◌	◌	◌	◌	◌	◌
Most researchers have no international research contacts	◌	◌	◌	◌	◌	◌
Most researchers are not internationally mobile	◌	◌	◌	◌	◌	◌
It is not possible to hire international researchers	◌	◌	◌	◌	◌	◌

	Strongly agree	Agree to some extent	Disagree to some extend	Strongly disagree	Do not know	N/A
Most researchers have to teach, and cannot put enough focus on their research	○	○	○	○	○	○
Most researchers have other jobs on the side and cannot put enough focus on their research	○	○	○	○	○	○

*30. What do you see as the main problems for research in your **country**?

	strongly agree	agree to some extent	neither agree nor disagree	disagree	strongly disagree	Do not know
Research is not given sufficiently high priority by the state	○	○	○	○	○	○
There is no national strategy, which would provide a clear framework	○	○	○	○	○	○
There is no or insufficient research funding	○	○	○	○	○	○
There are no or insufficient research facilities	○		○	○	○	○
Research is too much concentrated in a few institutions	○	○	○	○	○	○
Higher education institutions are focusing too much on teaching, and neglect research	○	○	○	○	○	○
Research is not sufficiently applied, and ignores the needs of the industry and society	○	○	○	○	○	○
There is too much focus on applied research	○	○	○	○	○	○
Interdisciplinary research is not sufficiently encouraged	○	○	○	○	○	○

	strongly agree	agree to some extent	neither agree nor disagree	disagree	strongly disagree	Do not know
The best researchers are leaving the country, which is a problem						
The best researchers are leaving the country, but they still continue to Contribute to national research						
Young researchers are leaving the country						
Young talented people look for careers outside of the research and higher Education sectors						

*31. What do you think are the reasons why people in your Institution are not, or limited engaged in research?

- Not enough time available to engage in research
- Clinical workload pressures
- Lack of support/encouragement
- Lack of funding
- Lack of available resources for research
- Difficulties gaining ethical approval
- Research not valued in organisation
- Weaknesses in research skills
- Personal circumstances (e.g., family commitments limiting time for research)
- Lack of research opportunities
- Lack of supervision/mentorship

☐ Difficulties collecting or gaining access to data

☐ There is no reason why people should be limited or not engaged in research

*32. What do you see as the main problems for research in your country?

	strongly agree	agree to some extent	neither agree nor disagree	disagree	strongly disagree	Do not know
Research is not given sufficiently high priority by the state	○	○	○	○	○	○
There is no national strategy, which would provide a clear framework	○	○	○	○	○	○
There is no or insufficient research funding	○	○	○	○	○	○
There are no or insufficient research facilities	○		○	○	○	○
Research is too much concentrated in a few institutions	○	○	○	○	○	○
Higher education institutions are focusing too much on teaching, and neglect research	○	○	○	○	○	○
Research is not sufficiently applied, and ignores the needs of the industry and society	○	○	○	○	○	○
There is too much focus on applied research	○	○	○	○	○	○
Interdisciplinary research is not sufficiently encouraged	○	○	○	○	○	○
The best researchers are leaving the country, which is a problem	○	○	○	○		○
The best researchers are leaving the country, but they still continue to Contribute to national research	○	○	○	○	○	○

	strongly agree	agree to some extent	neither agree nor disagree	disagree	strongly disagree	Do not know
Young researchers are leaving the country						
Young talented people look for careers outside of the research and higher Education sectors						

*33. Of the changes listed in the table below, indicate how effective you believe they would for increasing the level of research activity in your Institution

	Would have no effect	Would be somewhat effective	Would be greatly effective
Increased protected working time for research			
Higher levels of ongoing research training			
Recruiters in the health service giving more value to research experience when selecting candidates for clinical positions			
A more simplified and efficient ethics application process			
Greater levels of mentorship and support when conducting research			
More opportunities to advance one’s research qualifications (e.g. post-doctoral fellowships)			
More funded research posts			
More opportunities for collaboration with other clinical or academic researchers			

*34. What is applicable for you in the following list about **Research Infrastructure**.

	Don't know it	Know it	Collaborate with it
BBMRI Biobanking and Biomolecular Resources Research Infrastructure	◯	◯	◯
C-ERIC Central-European Research Infrastructures Consortium	◯	◯	◯
CLARIN Common Language Resources and Technology Infrastructure	◯	◯	◯
EATRIS European Advanced Translational Research Infrastructure in Medicine	◯	◯	◯
ECRIN European Clinical Research Infrastructure Network	◯	◯	◯
ELIXIR The European Life-Science Infrastructure for Biological Information	◯	◯	◯
ERA European Research Area	◯	◯	◯
ERIC European Research Infrastructure Consortium	◯	◯	◯
ESFRI European Strategy Forum on Research Infrastructure	◯	◯	◯
EU-OPENSCREEN European Infrastructure of Open Screening Platforms for Chemical Biology	◯	◯	◯
Euro-BioImaging European Research Infrastructure for Imaging Technologies in Biological and Biomedical Sciences	◯	◯	◯
INFRAFRONTIER European Research Infrastructure for the generation, phenotyping, archiving and distribution of mouse	◯	◯	◯
ISBE Infrastructure for Systems Biology Europe	◯	◯	◯
MIRRI Microbial Resource Research Infrastructure disease models	◯	◯	◯
NRRI Research Infrastructure Roadmap	◯	◯	◯

	Don't know it	Know it	Collaborate with it
PRACE Partnership for Advanced Computing in Europe	○	○	○
RI Research infrastructure(s)	○	○	○

*35. What is applicable for you in the following list about **Biobanks**.

	Don't know it	Know it	Collaborate with it
Basque Biobank	○	○	○
Biobanco.pt (Portuguese National Consortium of Biobanks)	○	○	○
Biobank Inherited Metabolic Disorders (blood/urine/liquor/fibroblasts)	○	○	○
Biobank of the Estonian genome center	○	○	○
BioNER: Biobank of the Institute of Rare Diseases Research/Institute of Health Carlos III	○	○	○
Brain-net – brain tissue bank Aachen	○	○	○
Brain-Net – brain tissue bank Munster (pediatric neuropathology)	○	○	○
Brain-Net Germany : National network of brain banks in Germany	○	○	○
Cell line and DNA Biobank from patients affected by genetic diseases	○	○	○
CIBERER Biobank (Biobank of rare diseases)	○	○	○
DNA biobank of the Department of Molecular Genetics and Diagnostics, National Institute of Environmental Health (NIEH) (EuroBioBank partner)	○	○	○
Genetic alliance Registry and Biobank	○	○	○

	Don't know it	Know it	Collaborate with it
EuroBioBank European Network of DNA, cell and tissue banks for Rare Diseases	◌	◌	◌
Human DNA and cell biobank of Genethon – genetic diseases, mainly neuromuscular diseases – part of the EuroBioBank network	◌	◌	◌
Human tissue biobank of Neuromuscular diseases (EuroBioBank partner)	◌	◌	◌
Neuromuscular Tissue Bank (NMTB) (EuroBioBank partner)	◌	◌	◌
Telethon Network of Genetic Biobanks	◌	◌	◌
UK Biobank	◌	◌	◌

*36. What is applicable for you in the following list about **Registry and database**.

	Don't know it	Know it	Collaborate with it
(NCARDRS) National Congenital Anomaly and Rare Disease Registration Service	◌	◌	◌
Austrian Country Node of the Human Variome Project (HVP)	◌	◌	◌
Austrian registry for inborn errors of metabolism	◌	◌	◌
Belgian Neuromuscular Disease Registry	◌	◌	◌
Belgian patient database for Wilson disease – contributes to the EuroWilson registry (terminated)	◌	◌	◌
Biobank Inherited Metabolic Disorders (blood/urine/liquor/fibroblasts)	◌	◌	◌
Canadian Neuromuscular Disease Registry – part of the TREAT-NMD network	◌	◌	◌

	Don't know it	Know it	Collaborate with it
DDRMD – Dutch Diagnosis Registration Metabolic Diseases	○	○	○
Dutch Neuromuscular Database CRAMP: Computer Registry of All Myopathies and Polyneuropathies	○	○	○
Dutch patient registry for Fabry disease	○	○	○
Dutch patient registry for Gaucher disease	○	○	○
Dutch patient registry for Niemann-Pick Disease Type B	○	○	○
Dutch patient registry for Niemann-Pick Disease Type C	○	○	○
ECARUCA: cytogenetic and clinical database on rare chromosomal disorders	○	○	○
E-HOD European Network and Registry for Homocystinurias and Methylation Defects	○	○	○
EIMD: European registry and network for intoxication type metabolic diseases	○	○	○
English alpha-1 antitrypsin deficiency registry – contributes to the Alpha One International Registry (AIR)	○	○	○
English cystinosis registry	○	○	○
English hyperoxaluria registry	○	○	○
English phenylketonuria registry	○	○	○
EPNET: European porphyria Network	○	○	○
E-RARE Consortium	○	○	○
ESNEK – Rare paediatric neurological disease registry Germany	○	○	○
EUNEFRON: registry of the European network for the study of orphan nephropathies	○	○	○
EUROCAT European Surveillance on congenital anomalies	○	○	○

	Don't know it	Know it	Collaborate with it
EUROGLYCANET – International patient registry and cohort for congenital disorders of glycosylation	◌	◌	◌
European cystinosis cohort	◌	◌	◌
European Porphyria Registry (EPR)	◌	◌	◌
Fabry Disease Registry –DE	◌	◌	◌
FOS : Fabry Outcome Survey	◌	◌	◌
French certified registry of glycogen storage disease type 2	◌	◌	◌
French certified registry of patients affected by Gaucher disease	◌	◌	◌
French cystinosis registry	◌	◌	◌
French registry of neuromuscular diseases from reference centres	◌	◌	◌
Galactosemia Patient Registry – part of the Galactosemia Network	◌	◌	◌
Genetic alliance Registry and Biobank	◌	◌	◌
German mucopolysaccharidosis patient registry	◌	◌	◌
HOS : Hunter Outcome Survey	◌	◌	◌
iNTD Network – International Working Group on Neurotransmitter Related Disorders	◌	◌	◌
International Collaborative Gaucher Group (ICGG) Gaucher Registry – DE	◌	◌	◌
International Morquio A registry	◌	◌	◌
International Niemann-Pick Disease Registry	◌	◌	◌
LOVD: GAA gene (Glucosidase, alpha, acid) variant database	◌	◌	◌
Mendelian cytogenetics network online database	◌	◌	◌

	Don't know it	Know it	Collaborate with it
MITOCON: National registry mitochondrial disease	○	○	○
Mucopolysaccharidosis type 1 (MPS I) Registry –	○	○	○
NCL-Registry: International neuronal ceroid lipofuscinoses patient registry	○	○	○
NCL-Registry: International neuronal ceroid lipofuscinoses patient registry	○	○	○
Neuro-Heart-Registry: Registry for patients with heart involment in neurovascular diseases	○	○	○
NMDcy: Cypriot Neuromuscular Diseases – contributes to TREAT-NMD	○	○	○
NMD-ES: Spanish Registry of Neuromuscular Diseases	○	○	○
Norvegian Porphyria Registry	○	○	○
Pompe Disease Registry – DE	○	○	○
Portuguese Fabry patient registry – contributing to the international Fabry registry	○	○	○
RAMEDIS : Rare Metabolic Diseases Database	○	○	○
RAMEDIS : Rare Metabolic Diseases mutation Database	○	○	○
Registry for Patients with Niemann-Pick Type C Disease	○	○	○
Registry for Wolfram syndrome, Alström syndrome, Bardet-Biedl syndrome and other rare diabetes syndromes – contributing to EURO-WABB	○	○	○
Registry_Biobank Name	○	○	○
The International Collaborative Gaucher Group (ICGG) Gaucher registry	○	○	○
The international Fabry registry	○	○	○
The international Mps I registry	○	○	○
The international Pompe registry	○	○	○

	Don't know it	Know it	Collaborate with it
TREAT-NMD: Accelerating Treatments for Neuromuscular Diseases (registries)	○	○	○
UK10K RARE NEUROMUSCULAR – Genetic neuromuscular diseases variant database	○	○	○
X-ALD: X-linked adrenoleukodystrophy database	○	○	○

*37. What is applicable for you in the following list about **Scientific Organization.**

	Don't know it	Know it	Collaborate with it
AECOM – Asociación Española para el Estudio de los Errores Congénitos del Metabolismo	○	○	○
APS – Arbeitsgemeinschaft für Pädiatrische Stoffwechselstörungen (Working group for paediatric metabolic disturbances, Germany)	○	○	○
B4B – Brains for Brain	○	○	○
BIMDG – British Inherited Metabolic Disease Group	○	○	○
CLIMB – Children Living with Inherited Metabolic Diseases, UK	○	○	○
EBC – European Brain Council	○	○	○
ERNDIM Quality assurance in laboratory testing for IEM	○	○	○
ESGCT – European Society of Gene and Cell Therapy	○	○	○
EURORDIS – European Organisation for Rare Disorders	○	○	○
Fondazione Telethon	○	○	○

	Don't know it	Know it	Collaborate with it
IBBS – International Brain Barriers Society			
IRDIRC			
ISNS – International Society for Neonatal Screening			
SEEIM – Sociedad Española de Errores Innatos del Metabolismo			
SFEIM – Societé Française pour l'Étude des Erreurs Innées du Métabolisme			
SIMD – Society for Inherited Metabolic Disorders			
SISMME – Societa' Italiana per lo Studio delle Malattie Metaboliche Ereditairie			
SPDM – Sociedade Portuguesa de Doenças Metabólicas			
SSIEM – The Society for Study of Inborn Errors of Metabolism			
